# From oral infection to pulmonary inflammation: periodontal pathogens and their potential role in COPD susceptibility

**DOI:** 10.3389/fcimb.2026.1896617

**Published:** 2026-08-28

**Authors:** Xiaohao Liu, Yan Chai, Fengxiang Shi, Bingchun Li, Jiawei Zeng, Jiaming Bi, Shuaimei Xu, Buling Wu

**Affiliations:** 1 Shenzhen Clinical College of Stomatology, School of Stomatology, Southern Medical University, Shenzhen, Guangdong, China; 2 Shenzhen Stomatology Hospital(Pingshan) of Southern Medical University, Shenzhen, Guangdong, China; 3 Department of Endodontics, Stomatological Hospital of Southern Medical University, Guangzhou, Guangdong, China; 4 Guangdong Provincial Center for Disease Control and Prevention, Guangzhou, China

**Keywords:** chronic obstructive pulmonary disease, inflammation, oral health, periodontal disease, periodontal pathogens

## Abstract

**Background:**

Oral health significantly impacts overall health. Periodontal disease and chronic obstructive pulmonary disease (COPD) are prevalent chronic inflammatory conditions among older adults, but their causal link is not well-established.

**Methods:**

Multivariable logistic regression, including subgroup, sex-stratified, and interaction analyses, was used to investigate the link between self-reported periodontal disease and COPD. Two-sample Mendelian randomization (MR) was utilized to explore causality, primarily through inverse-variance weighting (IVW), and supported by four additional MR approaches. Sensitivity analyses were performed to evaluate pleiotropy and heterogeneity. Normal human bronchial epithelial cells were stimulated *in vitro* with the periodontal pathogens *F. nucleatum* and *P. gingivalis*. The expression levels of interleukin-6 (IL-6) and interleukin-8 (IL-8) were then quantified using qPCR and chemiluminescence immunoassay. *In vivo*, a combined rat model of periodontitis and COPD was established to compare pulmonary inflammatory severity with a COPD-only model.

**Results:**

Multivariable logistic regression revealed significant associations between self-reported periodontal disease, oral hygiene practices, and COPD. The self-reported periodontal disease-COPD association was moderated by hypertension and flossing behavior. Mendelian randomization studies provided suggestive, exploratory evidence that periodontal disease may be associated with a higher risk of COPD in European populations, although the association did not survive multiple-testing correction and was not observed in East Asian populations. *In vitro*, both pathogens significantly upregulated IL-6 and IL-8 at both mRNA and protein levels. *In vivo*, rats with comorbid periodontitis and COPD exhibited significantly exacerbated pulmonary inflammation compared with those having COPD alone.

**Conclusion:**

Periodontal disease may promote COPD pathogenesis, with *in vitro* data supporting a direct pro-inflammatory role of *P. gingivalis* and *F. nucleatum* in airway epithelium. *In vivo*, periodontitis as a complex disease state exacerbates pulmonary inflammation in COPD. Maintaining good oral hygiene may represent a potential supportive strategy that warrants further investigation in interventional studies, with potential public health implications, especially among high-risk populations.

## Introduction

Periodontal disease and chronic obstructive pulmonary disease (COPD) are prevalent chronic inflammatory conditions globally, especially among the elderly, significantly impacting individual health and public healthcare systems (Scannapieco and Ho, 2001; Scannapieco and Cantos, 2016). Since the conceptual emergence of “Periodontal Medicine” in the 1990s, the bidirectional interplay between oral infections and systemic pathologies has garnered increasing scientific scrutiny (Beck et al., 2019). Acting as a primary gateway to the lower respiratory tract, the oral cavity is also a significant ecological niche for microbes, may exert pathological effects on distant organ systems, particularly under the chronic inflammatory condition of periodontal disease. This effect occurs through mechanisms that involve the spread of oral pathogens via the bloodstream and the release of pro-inflammatory substances throughout the body. The potential link between periodontal disease and COPD has gained considerable interest in the combined research fields of respiratory medicine and oral biology (Baker et al., 2024). Emerging evidence suggests that oral diseases, particularly periodontal disorders, may affect the development and outcome of several systemic illnesses, including those related to the respiratory, cardiovascular, cerebrovascular, and immune systems (Kapila, 2021; Botelho et al., 2022; Peng et al., 2022; Deng et al., 2025).

COPD is a prevalent chronic inflammatory respiratory disorder, posing a significant global public health challenge and impacting around 300 million people worldwide (Christenson et al., 2022). Inflammatory processes play an important part in the emergence of periodontal disease and COPD in older adults. In patients with COPD, coexisting periodontal disease may increase systemic inflammation, potentially influencing disease progression (Sapey et al., 2020; Qi et al., 2024). Emerging evidence indicates a clear connection between periodontal disease and COPD (Lin et al., 2023; Wang et al., 2024). A Mendelian randomization study has determined a significant genetically predicted causal link, indicating that periodontitis is an independent risk factor for COPD (Zhao et al., 2024). Furthermore, several clinical observations have documented that individuals with COPD frequently present with compromised periodontal health, manifesting as increased probing pocket depths and greater clinical attachment loss (Satyanarayana et al., 2023).

While observed, the exact cause-and-effect link between periodontal disease and COPD remains unclear, requiring more in-depth research. Recent studies indicate that periodontal disease and COPD share risk factors, which include demographic and behavioral aspects like gender, age, ethnicity, tobacco and alcohol use, along with metabolic and cardiovascular comorbidities such as diabetes, obesity, and hypertension (Qiu et al., 2022; Selvaraj et al., 2022; Grant et al., 2023; Satyanarayana et al., 2023; Du et al., 2024). Extant epidemiological studies, however, are often constrained by limitations in sample representativeness and incomplete adjustment for confounding variables. The integration of multidimensional behavioral and socioeconomic covariates into analytical frameworks continues to demand larger-scale, more robust evidence to substantiate reported associations.

With this background, the study seeks to systematically examine the association between self-reported periodontal disease and COPD through a cost-effective approach that aims to minimize residual confounding. The purpose of our study is to analyze the association between self-reported periodontal disease and COPD using NHANES data and to identify causal directionality via Mendelian randomization analysis. *In vitro* cellular models and *in vivo* animal experiments will be used to validate the mechanistic role of periodontal disease in COPD pathogenesis, providing biological plausibility and empirical support for the population-level findings. Exploring the connection between periodontal health, oral health, and COPD, along with assessing the impact of oral health interventions on COPD management, can provide strategies to lessen disease burden and enhance quality of life, providing a new approach for integrated COPD prevention and management.

## Methods

### A cross-sectional analysis utilizing the NHANES database

#### Study cohort and data origin

NHANES conducts a cross-sectional analysis of the health and nutrition of non-institutionalized civilians in the U.S. by means of extensive household interviews, standardized physical evaluations, and laboratory testing. The NHANES protocol was ethically approved by the NCHS Research Ethics Review Board, and written informed consent was obtained from all participants prior to their involvement.

**Figure 1:**
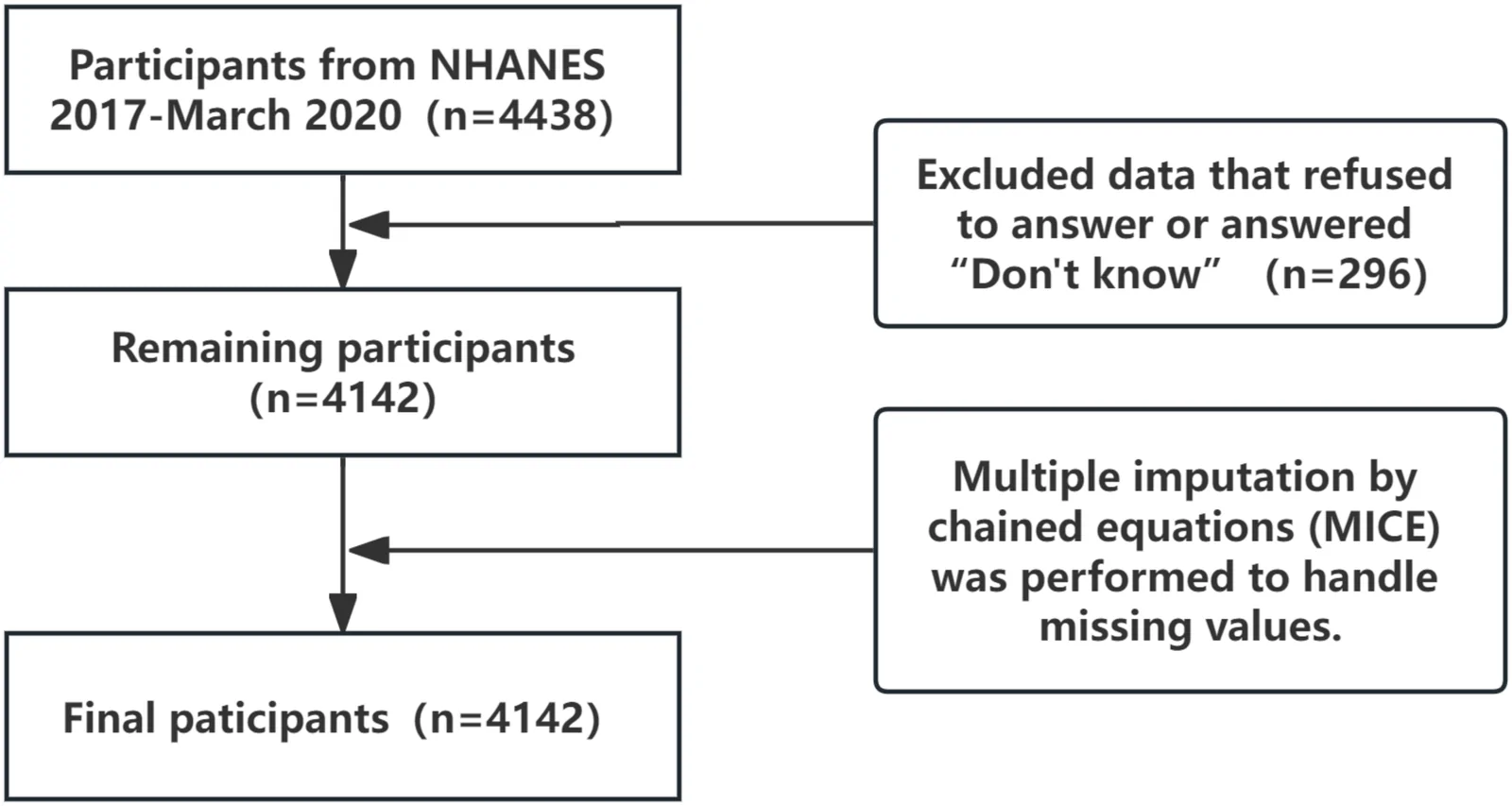
Flow chart of procedures from identification of eligible patients to final inclusion.

This study utilized data from the NHANES 2017–March 2020 pre-pandemic cycle to investigate the relationships between COPD, periodontal disease, oral health status, and oral hygiene behaviors. Responses recorded as “Refused” or “Don’t know” were excluded from the analytical dataset. Missing values were addressed by multiple imputation using chained equations (MICE) in accordance with the study’s prespecified methodological framework (**Figure 1**). We performed MICE imputation on all original variables to generate 20 complete datasets (with seed = 666 for reproducibility). For continuous variables such as age, BMI, and hsCRP, we applied predictive mean matching; for binary variables including smoking status and hypertension, we used logistic regression; and for categorical variables with more than two levels, such as race/ethnicity and education level, we employed multinomial logistic regression. To assess convergence, we visually inspected trace plots of the mean and standard deviation of imputed values across iterations using the “plot() function” in the “mice” package. Convergence was further evaluated using the R-hat (potential scale reduction factor), calculated with the “Rhat.mice() function” from the “miceadds” package. To compare observed and imputed distributions, we generated density plots using the “densityplot() function” in the “mice” package. After imputation, categorical variables derived from continuous measures were derived from the imputed continuous data, such as age groups (<60 vs. ≥60 years), poverty-to-income ratio categories (<1.3, 1.3–3.5, >3.5), BMI categories, hsCRP categories and so on. These 20 datasets were then combined into a single complete dataset according to established pooling principles. Descriptive statistics (including means and proportions) were calculated from the pooled dataset. For regression analyses (including multivariable analysis and interaction analysis), models were fitted separately in each of the 20 imputed datasets, and the results were combined using Rubin’s rules. Additionally, variables with >20% missingness were reviewed for potential exclusion. The final analytic sample after multiple imputation comprised 4,142 participants. The missing proportions for all variables are presented in **Supplementary Table 2**.

### Evaluation of COPD, periodontal disease, oral health, and hygiene practices

COPD status was identified based on participants’ affirmative response to the survey question: “Has a doctor or other health professional ever told you that you had chronic obstructive pulmonary disease (COPD)?” This measure reflects self-reported physician-diagnosed COPD, rather than spirometry-confirmed COPD. Periodontal disease status was assessed using the self-report question: “Do you think you might have gum disease?” This question captures participants’ perceived gum health rather than clinically confirmed periodontitis, with affirmative responses indicating self-reported periodontal disease.

Oral health was assessed through the self-evaluation question, ‘Overall, how would you rate the health of your teeth and gums?’ Responses were collected using a five-point Likert scale: Excellent, Very Good, Good, Fair, and Poor. For analysis, responses labeled as Excellent, Very Good, and Good were merged into a single category called ‘Good,’ while Fair and Poor responses were grouped under ‘Poor’.

Flossing frequency was derived from the question: “In the past week, on how many days did you use dental floss or any other device to clean between your teeth?” Participants reporting zero days were classified as “No flossing,” while those reporting one to seven days were categorized as “Flossing”.

### Covariates

The analytical models included a wide range of covariates: demographic factors (sex, age, race/ethnicity, education level, poverty-to-income ratio, and BMI), lifestyle behaviors (smoking and alcohol use), comorbidities (hypertension, hyperlipidemia, and diabetes), and serum hsCRP levels. Comorbidity data were derived from self-reported questionnaire responses collected during the NHANES 2017–March 2020 pre-pandemic cycle. Never smokers are defined as people who have smoked under 100 cigarettes throughout their lifetime. Alcohol consumption was described as the intake of at least 12 ounces of beer, 5 ounces of wine, or 1.5 ounces of distilled spirits. The World Health Organization (WHO) standards were used to classify body mass index into obesity, overweight, normal weight, and underweight. Serum hsCRP levels were stratified into low-, intermediate-, and high-risk categories based on the risk thresholds established by the American Heart Association (AHA).

### Statistical analysis

All regression analyses incorporated NHANES sampling weights, strata, and primary sampling units (PSUs) to account for the complex multistage stratified cluster design. For the 2017–March 2020 pre-pandemic cycle, the appropriate MEC examination weight is WTMECPRP (rather than the standard 2 year WTMEC2YR), as this weight has been specifically calibrated for the 39 month pre-pandemic sample. The survey design was specified using the “svydesign() function” in R, with id = ~SDMVPSU, strata = ~SDMVSTRA, and weights = ~WTMECPRP. The research employed multivariable logistic regression models to investigate the links between COPD and self-reported periodontal disease, oral health status, and oral hygiene routines. Categorical variables were expressed as weighted percentages with standard errors, while continuous variables were expressed as means with standard deviations. To compare these between groups, chi-square tests, Wilcoxon rank-sum tests, or t-tests were applied, as suitable. The analytical findings are presented across four sequentially adjusted models. Model 1 was adjusted for sex, age, and race/ethnicity. Model 2 included additional adjustment for educational attainment and poverty-to-income ratio. Model 3 further incorporated lifestyle covariates, namely smoking status and alcohol consumption. Model 4 was fully adjusted for all covariates specified in the preceding section. Analyses of subgroups and interactions were performed to explore possible effect modifications across various population segments. Statistical analyses were conducted with R software (v4. 3. 2) and the EmpowerStats package (v5. 0) to maintain analytical rigor and reproducibility.

### Mendelian randomization analysis

#### Principles of mendelian randomization

**Figure 2:**
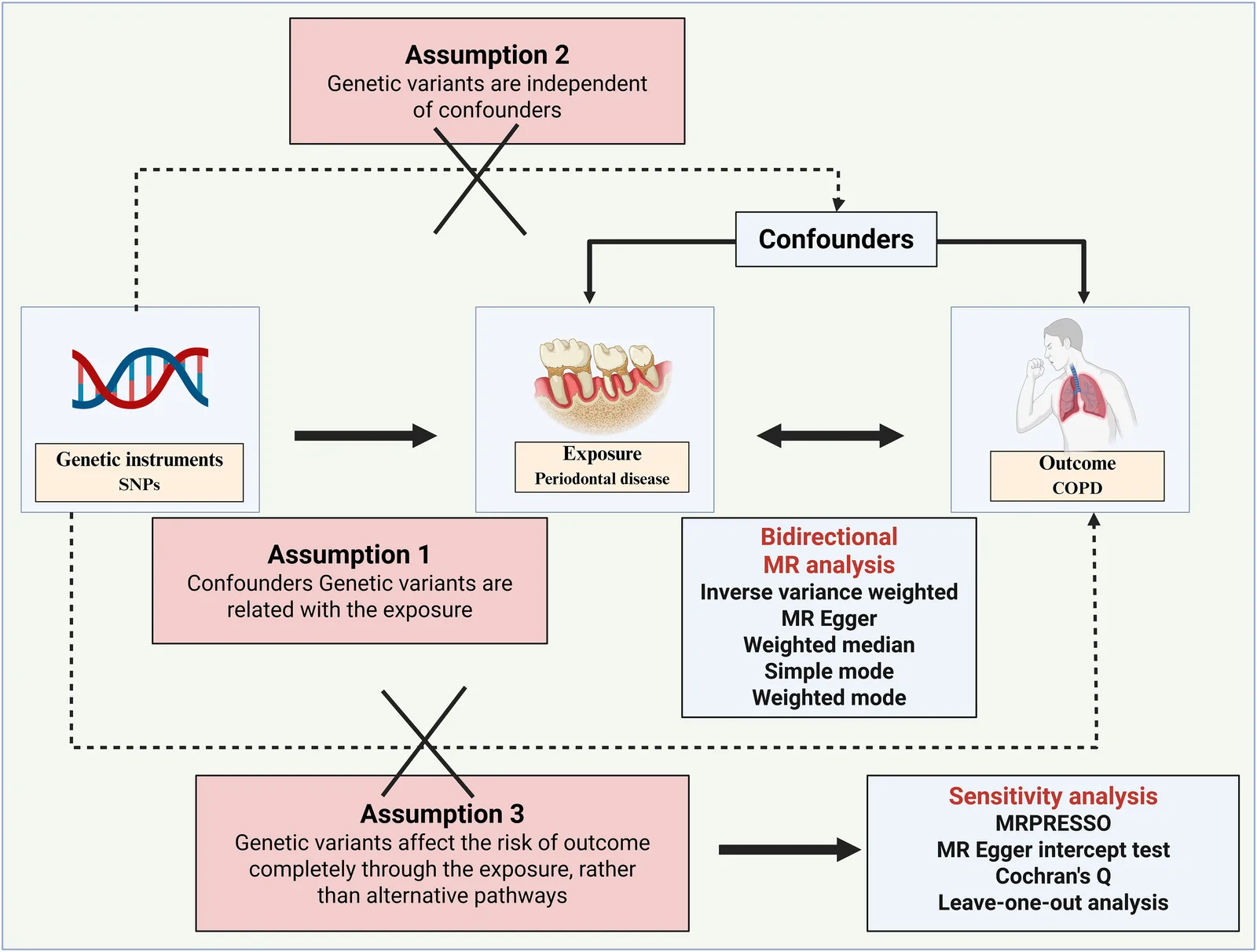
Schematic flow chart of two-way Mendelian analysis.

Mendelian randomization (MR) is a causal inference method using genetic variants as instrumental variables, based on Mendel’s laws of segregation and independent assortment, to mimic randomized controlled trial conditions. This study used a two-sample Mendelian Randomization (MR) approach to assess the causal impact of exposure on the outcome, employing summary-level genome-wide association study (GWAS) data from two separate cohorts. The MR approach is valid if three key assumptions are met: (i) the genetic instrument must be strongly linked to the exposure; (ii) it should be independent of any confounders affecting the exposure-outcome relationship; and (iii) it should impact the outcome solely through the exposure, without any pleiotropic effects. Bidirectional two-sample MR analyses were performed to explore the potential causal link between self-reported periodontal disease and COPD (**Figure 2**).

#### Data sources and instrumental variable selection

All MR analyses utilized publicly available GWAS summary statistics from ethically approved studies with informed consent from participants. GWAS data for COPD and periodontal disease were retrieved from the GWAS Catalog and the National Bioscience Database Center (NBDC). The European COPD GWAS (GCST90399694) was derived from the Global Biobank Meta-analysis Initiative, using clinically characterized COPD cases (Zhou et al., 2022). The European periodontal disease GWAS (GCST008441) was based on clinically assessed periodontal complex traits, including probing depth and clinical attachment loss (Offenbacher et al., 2016). The East Asian periodontal disease GWAS (GCST90018677) was based on electronic health record-defined periodontal disease from the Pan-UKBB East Asian dataset (Sakaue et al., 2021). The East Asian COPD GWAS (hum0014) was derived from BioBank Japan, using ICD-coded diagnoses (Ishigaki et al., 2020). The sample sizes for each dataset were as follows: European COPD: 58,559 cases and 937,358 controls (total n = 995,917); European periodontal disease: 3,569 cases and 6,124 controls (total n = 9,693); East Asian COPD: 3,315 cases and 201,592 controls (total n = 204,907); East Asian periodontal disease: 9,560 cases and 169,166 controls (total n = 178,726). **Supplementary Table 6** offers comprehensive details of the GWAS datasets used in this analysis. Some single nucleotide polymorphisms (SNPs) were significantly associated with the exposure phenotype across the genome, with a P-value less than 5×10^-6^, were identified as instrumental variables. Genetic instruments were ensured to be independent by performing linkage disequilibrium clumping with a strict threshold of r²<0. 001 within a 10,000 kb window. Instrumental variable strength was measured by the F-statistic, and SNPs with an F-statistic lower than 10 were omitted to avoid bias from weak instruments in later analyses. SNP harmonization was used to ensure effect alleles were consistent between the exposure and outcome datasets, removing palindromic and incompatible SNPs from the analysis.

#### Statistical and sensitivity analyses

The primary analytical approach for estimating the causal effect of periodontal disease on COPD was the inverse variance weighted (IVW) method. To improve the robustness and reliability of causal estimates, four complementary MR methods — MR-Egger, weighted mode, weighted median, and simple mode were utilized to manage potential violations of MR assumptions, including directional pleiotropy and instrument heterogeneity (Bowden et al., 2016). A comprehensive suite of sensitivity analyses was performed to evaluate the validity of the MR findings. The MR-Egger regression intercept test was used to evaluate horizontal pleiotropy, where a non-significant intercept (*P > 0.05*) suggests no significant directional pleiotropy. Cochran’s Q statistic was applied to determine heterogeneity among causal estimates from individual SNPs, with a P value higher than 0.05 indicating no significant heterogeneity. The impact of each genetic variant on the causal estimate was evaluated using a leave-one-out sensitivity analysis, which also aimed to detect any outlier SNPs. The R software (version 4.3.2) with TwoSampleMR and MR-PRESSO packages was used to perform Mendelian randomization analyses. Scatter and forest plots were used for result visualization.

### Bacterial culture

The *F. nucleatum* strain BNCC337608 and *P. gingivalis* strain 83W were inoculated into Brain Heart Infusion broth and incubated anaerobically for 24 hours at 37 °C with a gas mixture of 85% N_2_, 10% H_2_, and 5% CO_2_. The bacterial culture was transferred to fresh BHI broth and incubated under identical conditions until reaching the mid-logarithmic growth phase for further experiments.

### Cell culture

BEAS-2B cells were incubated at 37 °C in DMEM (Gibco, Carlsbad, CA, USA) with 10% fetal bovine serum, 100 U/ml penicillin, and 100 μg/ml streptomycin, in a 5% CO_2_ atmosphere. For the experiments, BEAS-2B cells were made confluent by seeding 24-well plates with 2.5 × 10^5^ cells per well and leaving them overnight. Cells were washed and the medium replaced with serum-free DMEM prior to adding *F. nucleatum* and *P. gingivalis*. BEAS-2B cells were exposed to varying concentrations of *F. nucleatum* and *P. gingivalis* for 24 hours, with phosphate-buffered saline serving as the control. The multiplicity of infection (MOI) was defined as the ratio of viable bacteria to target cells.

### CCK-8 cell viability assay

To assess the potential cytotoxic effects of bacterial exposure on BEAS-2B cells, cell viability was evaluated using the Cell Counting Kit-8 (CCK-8, Beyotime Biotechnology, Shanghai, China) according to the manufacturer’s instructions. Briefly, BEAS-2B cells were seeded into 96-well plates at a density of 5.0 × 10^3^ cells per well and cultured overnight. Since live bacteria interfere with the absorbance readings of the CCK-8 assay, we used bacterial culture supernatants rather than live bacteria in the CCK-8 experiments to avoid this interference. After 24 hours of treatment with bacterial culture supernatants, the culture medium was replaced with 100 μL of fresh medium containing 10 μL of CCK-8 solution. After incubation at 37 °C for 2 hours, the absorbance at 450 nm (OD_450_) was measured using a microplate reader (Bio-Tek Instruments, Winooski, VT, USA). Cell viability was calculated as the percentage of the control group (PBS-treated cells, set at 100%).

### Quantitative real-time polymerase chain reaction

AG RNAex Pro RNA Extraction Reagent (Aikerui Bio, Hunan, China) was used to isolate total RNA from mouse lung tissues and cultured cells. RNA samples were reverse-transcribed with the Evo M-MLV Reverse Transcription Premixed Kit (Aikerui Bio, Hunan, China). The primer sequences for gene amplification were: human IL6 (forward: 5’-ATAACCACCCCTGACCCAAC-3’, reverse: 5’-CCCATGCTACATTTGCCGAA-3’), human IL8 (forward: 5’-TCAGAGACAGCAGAGCACAC-3’, reverse: 5’-GGCAAAACTGCACCTTCACA-3’), human GAPDH (forward: 5’-ACCAGCCCCAGCAAGAGCACAAG-3’, reverse: 5’-TTCAAGGGGTCTACATGGCAACTG-3’). cDNA amplification and detection were performed using a Roche LightCycler 96 system (Roche Diagnostics, Basel, Switzerland) with a SYBR Green Pro Taq HS Premixed qPCR Kit (Aikerui Bio, Hunan, China). Gene expression levels were quantified using the ΔΔCt method.

### Chemiluminescence immunoassay

BEAS-2B cells were exposed to *P. gingivalis* or *F. nucleatum* at an MOI of 100 for 24 hours. After cells were co-cultured, the supernatants were collected and centrifuged at 12,000 × g for 10 minutes at 4 °C to clear out cellular debris. After clarification, the supernatants were divided into aliquots and either analyzed on the spot or stored at -80 °C to avoid freeze-thaw cycles. The measurements were performed using the Servicebio^®^ Human IL-6 MPCLIA Kit (Servicebio, Wuhan, China)and the Servicebio^®^ Human IL-8 MPCLIA Kit (Servicebio, Wuhan, China). In summary, 25 µL of either supernatant or standard was introduced to antibody-coated microwells and incubated at 37 °C for 60 minutes. Following washing, a detection antibody conjugated with horseradish peroxidase was added and incubated at 37 °C for 30 minutes. Following thorough washing to remove unbound conjugates, a chemiluminescent substrate was added. After 5 minutes of reaction, the relative light unit values were measured immediately using a chemiluminescence immunoassay analyzer H-150 (Servicebio, Wuhan, China). The instrument’s software automatically generated a four-parameter logistic standard curve using the RLU values from the serial dilutions of the standards, with ranges of 10 to 10000 pg/mL for IL-6 and 25 to 1600 pg/mL for IL-8. The concentration of each sample was interpolated from this standard curve. Samples yielding values outside the standard curve range were appropriately diluted with the provided diluent and re-analyzed. All samples were assayed in triplicate.

### *In Vivo* rat model

Animal experiments adhered to the Helsinki Declaration guidelines for research involving animals and was granted approval by the Rege Biotechnology Laboratory Animal Ethics Committee (Approval No: 20250528-001). SPF male Sprague-Dawley rats were obtained from RuiGe Biological Technology Co. Ltd. in Guangzhou, China. A total of 18 male Sprague-Dawley rats were randomly assigned to three groups (n = 6 per group): (1) normal control group (PBS instillation), (2) COPD-only group (LPS instillation + cigarette smoke exposure), and (3) COPD + periodontitis group (ligature-induced periodontitis with *P. gingivalis* and *F. nucleatum* inoculation, combined with LPS instillation and cigarette smoke exposure). Sample size was determined based on a power calculation assuming an effect size of 1.5, α = 0.05, and power = 0.80. No animals were excluded from the analysis. The subjects were kept in a controlled environment (22 ± 2 °C temperature, 50 ± 10% humidity, 12-hour light/dark cycle) with unrestricted access to standard rodent chow and water. Rats aged three weeks received anesthesia through an intraperitoneal injection of sodium pentobarbital at a dose of 40 mg/kg. Periodontitis was induced by ligating the cervical region of the bilateral maxillary first molars with sterile 5–0 silk sutures, which were retained in the gingival sulcus to promote local plaque accumulation. To increase plaque pathogenicity, a mixture of *P. gingivalis* and *F. nucleatum* (2 × 10^8^ CFU/mL, 50 µL each) was applied to the oral cavity around the ligated areas three times weekly for 3 weeks using a sterile cotton swab, starting from the day of ligation. Sham-operated rats received anesthesia and application of sterile saline only, without ligation. Starting at six weeks of age, a COPD model was superimposed on the periodontitis model. Rats received intratracheal instillation of lipopolysaccharide (LPS, 1 mg/kg body weight dissolved in sterile saline) once every two weeks, commencing at 6 weeks of age and continuing until one week prior to sacrifice. Concurrently, from the initiation of LPS administration until the experimental endpoint (12 weeks of age), rats were exposed to cigarette smoke daily. The exposure was conducted in a custom-made acrylic chamber using commercial cigarettes (Hongshuangxi, China) as the smoke source. Rats were exposed twice daily (with an interval of at least 4 hours between sessions), with each session lasting 30 minutes, consuming an average of 3 cigarettes per rat per day. All rats were sacrificed at 12 weeks of age. After administering a deep anesthesia overdose of sodium pentobarbital, the thoracic cavity was opened, and the lungs were meticulously dissected. The left lung was lavaged with pre-cooled saline and then immediately fixed in 4% paraformaldehyde phosphate buffer for histological analysis.

### Histological processing and hematoxylin-eosin staining

The lung tissues underwent a 48 hour fixation, were dehydrated using graded ethanol, cleared with xylene, and embedded in paraffin wax. Coronal sections, each 5 µm thick, were cut serially with a microtome. After removing paraffin and rehydrating, the sections were processed with standard hematoxylin and eosin (H&E) staining: hematoxylin for 5 minutes, followed by rinsing in running tap water for bluing, and counterstaining with eosin for 30–60 seconds. Sections underwent dehydration and clearing before being mounted with neutral balsam. Stained sections were initially examined under an upright light microscope (Leica DMi8, Leica Microsystems, Germany). Representative fields of view were captured at magnifications of × 100 and × 200 using the microscope-integrated digital camera system and the manufacturer’s image acquisition software (Leica Application Suite X, LAS X). To provide quantitative support for histopathological observation, we adopted a semi quantitative pulmonary inflammation scoring system evaluating three parameters on a 0–4 scale (The scoring criteria are detailed in **Supplementary Table 13**): (1) alveolar wall thickening, (2) inflammatory cell infiltration, and (3) alveolar structural disruption. The total score ranged from 0 to 12. Scoring was performed independently by two pathologists blinded to group allocation, and the average score was used for analysis (intraclass correlation coefficient > 0.85).

### Bronchoalveolar lavage fluid collection and analysis

Following sacrifice, the left lung was lavaged with 1 mL of pre-cooled sterile phosphate-buffered saline (PBS) via a tracheal cannula. The lavage fluid was gently aspirated and reinjected three times to maximize recovery. The recovered BALF was centrifuged at 1,500 × g for 10 minutes at 4 °C, and the supernatant was collected for protein concentration measurement using the bicinchoninic acid (BCA) protein assay kit (Beyotime Biotechnology, Shanghai, China), following the manufacturer’s protocol. The cell pellet was resuspended in PBS, and total cell counts were determined using a hemocytometer. To minimize inter-animal variability due to differences in lavage fluid recovery, we performed relative quantification of total protein concentration and total cell counts. In the relative quantification analysis, the normalized values from each experimental group were expressed as fold-change relative to the normal control group (which was set to 1.0), allowing for direct comparison of pulmonary inflammation severity across the three groups.

### Micro-CT validation of periodontitis induction

To confirm successful periodontitis induction, maxillary alveolar bone loss was assessed using micro-computed tomography (micro-CT). After sacrifice, rat maxillae were dissected and fixed in 4% paraformaldehyde for 48 hours. Specimens were scanned using a micro-CT system (SkyScan 1176, Bruker, Kontich, Belgium). Three-dimensional reconstructions were performed using NRecon software (Bruker, Kontich, Belgium). The distance from the cemento-enamel junction (CEJ) to the alveolar bone crest (ABC) was measured on the mesial aspect of the maxillary first molars using CTAn software (Bruker, Kontich, Belgium). Three consecutive sections were measured per tooth, and the average value was calculated for each animal.

### Statistical analysis

Each experiment was conducted with a minimum of three independent replicates. Control groups received phosphate-buffered saline (mock treatment) and mock treatment. Data are shown as mean ± standard deviation (SD). GraphPad Prism (version 10. 0) was used for data visualization, and SPSS (version 20. 0) was employed for statistical analysis. To compare two groups, the Student’s t-test was used, while a one-way ANOVA followed by Tukey’s *post hoc* test was employed for multiple group comparisons. Statistical significance was assigned to p-values under 0.05.

## Results

### Convergence diagnostics of multiple imputation

The trace lines showed no trend and were well mixed after 20 iterations, providing evidence that the algorithm had converged satisfactorily (**Supplementary Figure 6**). **Supplementary Table 2** provides the missing proportion, along with the mean and variance R-hat estimates for each variable. For variables without missing data, R-hat was not applicable and is therefore indicated as NA. All R-hat values for imputed variables were below or equal to 1.09, confirming that the imputation algorithm had adequately converged. Density plots comparing observed and imputed distributions has shown that the imputed distributions closely overlap with the observed distributions, indicating that the imputation model produced plausible values (**Supplementary Figure 7**).

### NHANES participants’ demographic and health characteristics

The research included 4,142 participants from the NHANES 2017–March 2020 pre-pandemic cycle to examine the association between COPD and periodontal disease. **Table 1**, **Supplementary Table 1** present the baseline demographic, lifestyle, oral health, and clinical characteristics of the study cohort, categorized by periodontal disease and COPD status. The average age of participants was 49.46 years (SD = 18.29), with 34.7% (n = 1,438) being 60 years or older. The cohort included 1,991 males and 2,151 females. With respect to racial and ethnic composition, 33.9% of participants identified as non-Hispanic White people. Regarding socioeconomic indicators, 57.4% of participants had attained at least a college education, and 29.8% reported a poverty-to-income ratio of ≥ 3.5. Obesity prevalence, indicated by a BMI of 30 kg/m² or higher, was 41.1%.

**Table 1:** The baseline characteristics of participants with or without COPD in a cross-sectional study based on the NHANES 2017-March 2020 Pre-pandemic.

Variables	Overall (n=4142)	COPD No (n=3776) Yes (n=366)		P-value
Periodontal disease, n (%)				< 0.001
Yes	713 (17.2%)	603 (16.0%)	110 (30.1%)	
No	3429 (82.8%)	3173 (84.0%)	256 (69.9%)	
Oral health, n (%)				< 0.001
Good	2758 (66.6%)	2560 (67.8%)	198 (54.1%)	
Poor	1384 (33.4%)	1216 (32.2%)	168 (45.9%)	
Age, Mean ± SD	49.46 ± 18.29	48.39 ± 18.23	60.53 ± 14.94	< 0.001
Age strata, n (%)				< 0.001
<60	2704 (65.3%)	2560 (67.8%)	144 (39.3%)	
≥60	1438 (34.7%)	1216 (32.2%)	222 (60.7%)	
Gender, n (%)				0.035
Male	1991 (48.1%)	1823 (48.3%)	168 (45.9%)	
Female	2151 (51.9%)	1953 (51.7%)	198 (54.1%)	
Race, n (%)				< 0.001
Non-Hispanic White	1405 (33.9%)	1209 (32.0%)	196 (53.6%)	
Mexican American	505 (12.2%)	488 (12.9%)	17 (4.6%)	
Other Hispanic	399 (9.6%)	374 (9.9%)	25 (6.8%)	
Non-Hispanic Black	1093 (26.4%)	1006 (26.6%)	87 (23.8%)	
Non-Hispanic Asian	517 (12.5%)	510 (13.5%)	7 (1.9%)	
Other Race	223 (5.4%)	189 (5.0%)	34 (9.3%)	
Education level, n (%)				< 0.001
Under high school	760 (18.3%)	683 (18.1%)	77 (21.0%)	
High school/GED or equivalent	1004 (24.2%)	883 (23.4%)	121 (33.1%)	
Above high school	2378 (57.4%)	2210 (58.5%)	168 (45.9%)	
Income-to-poverty ratio, n (%)				< 0.001
<1.3	1156 (27.9%)	1023 (27.1%)	133 (36.3%)	
1.3-3.5	1753 (42.3%)	1571 (41.6%)	182 (49.7%)	
>3.5	1233 (29.8%)	1182 (31.3%)	51 (13.9%)	
Alcohol use, n (%)				0.006
Yes	3763 (90.8%)	3411 (90.3%)	352 (96.2%)	
No	379 (9.2%)	365 (9.7%)	14 (3.8%)	
Smoking, n (%)				< 0.001
Yes	1687 (40.7%)	1414 (37.4%)	273 (74.6%)	
No	2455 (59.3%)	2362 (62.6%)	93 (25.4%)	
Dental floss usage				< 0.001
Yes	3016 (72.8%)	2827 (74.9%)	189 (51.6%)	
No	1126 (27.2%)	949 (25.1%)	177 (48.4%)	
BMI, n (%)				< 0.001
Underweight	72 (1.7%)	59 (1.6%)	13 (3.6%)	
Normal weight	1051 (25.4%)	987 (26.1%)	64 (17.5%)	
Overweight	1318 (31.8%)	1218 (32.3%)	100 (27.3%)	
Obesity	1701(41.1%)	1512 (40.0%)	189 (51.6%)	
Hypertension, n (%)				< 0.001
Yes	1552 (37.5%)	1333 (35.3%)	219 (59.8%)	
No	2590 (62.5%)	2443 (64.7%)	147 (40.2%)	
Hyperlipidemia, n (%)				< 0.001
Yes	1455 (35.1%)	1272 (33.7%)	183 (50.0%)	
No	2687 (64.9%)	2504 (66.3%)	183 (50.0%)	
Diabetes, n (%)				< 0.001
Yes	636 (15.4%)	527 (14.0%)	109 (29.8%)	
Borderline	123 (3.0%)	107 (2.8%)	16 (4.4%)	
No	3383 (81.7%)	3142 (83.2%)	241 (65.8%)	
Fasting Glucose (mmol/L), Mean ± SD	6.24 ± 2.01	6.17 ± 1.91	6.89 ± 2.78	< 0.001
Glycohemoglobin (%), Mean ± SD	5.84 ± 1.11	5.8 ± 1.08	6.24 ± 1.30	< 0.001
hsCRP (mg/L), Mean ± SD	4.01 ± 7.38	3.77 ± 6.71	6.47 ± 12.09	< 0.001
hsCRP (mg/L), n (%)				< 0.001
Low risk	1250 (30.2%)	1190 (31.5%)	60 (16.4%)	
Medium risk	1394 (33.7%)	1293 (34.2%)	101 (27.6%)	
High risk	1498 (36.2%)	1293 (34.2%)	205 (56.0%)	

Within the analytical sample, self-reported periodontal disease was documented in 17.2% (n = 713) of participants, whereas COPD was present in 8.8% (n = 366). Significant differences were found between participants with and without periodontal disease in terms of sex, education level, poverty-to-income ratio, smoking habits, self-assessed oral health, and the prevalence of hypertension, diabetes mellitus, and hyperlipidemia (all *P<0.05*). Participants diagnosed with COPD were significantly more likely to be aged 60 years or older, corroborating an age-dependent susceptibility to the disease. Individuals with COPD showed significantly higher levels of fasting glucose, glycated hemoglobin (HbA1c), and high-sensitivity C-reactive protein (hsCRP) compared to those without COPD (all *P < 0.05*).

### Correlation between COPD and periodontal disease alongside oral health factors

Multivariable logistic regression analyses were performed across four sequentially adjusted models to assess the relationships between COPD and oral health parameters (**Table 2**). In Models 1 to 3, which progressively adjusted for sociodemographic and behavioral confounders such as age, sex, race, educational attainment, poverty-to-income ratio, smoking status, and alcohol consumption, COPD consistently showed a significant association with self-reported periodontal disease, self-rated oral health status, and oral hygiene behaviors. Model 4 revealed a significant positive correlation between COPD and self-reported periodontal disease (OR = 2.160, 95% CI: 1.308 to 3.565, *P = 0. 024)*. Regular flossing (OR = 0.472, 95% CI: 0.380 to 0.585, *P* < *0.001*) was significantly inversely associated with COPD. The link between COPD and self-rated oral health status weakened and statistical significance was not maintained in the fully adjusted model (OR = 1.669, 95% CI: 1.066 to 2.614, *P = 0.066*). These findings demonstrate a strong positive correlation between self-reported periodontal disease and COPD, while regular flossing are significantly negatively correlated with COPD. Poorer self-rated oral health demonstrated a non-significant positive trend with COPD.

**Table 2:** Associations between periodontal disease and COPD in NHANES 2017-March 2020 Pre-pandemic according to multivariable logistic regression analysis. .

Variables	Model 1 OR (95% CI) P-value		Model 2 OR (95% CI) P-value		Model 3 OR (95% CI) P-value		Model 4 OR (95% CI) P-value	
Periodontal disease	2.653 (1.532, 4.595)	0.003	2.497 (1.500, 4.156)	0.004	2.331 (1.415, 3.839)	0.007	2.160 (1.308, 3.565)	0.024
Oral health	2.604 (1.697, 3.995)	< 0.001	2.013 (1.293, 3.133)	0.008	1.770 (1.129, 2.775)	0.030	1.669 (1.066, 2.614)	0.066
Dental floss usage	0.351 (0.284, 0.433)	< 0.001	0.439 (0.360, 0.536)	< 0.001	0.461 (0.367, 0.580)	< 0.001	0.472 (0.380, 0.585)	< 0.001

Model 1, Adjusting for age, gender, race;.

Model 2, Model 1 + adjusted for educational level, ratio of family income to poverty.

Model 3, Model 2 + adjusted for smoking and alcohol use.

Model 4, Model 3+ adjusted for BMI, hypertension, hyperlipidemia, diabetes, and hsCRP.

OR, Odds ratio; CI, Confidence interval.

### Subgroup analyses and effect modification

**Figure 3:**
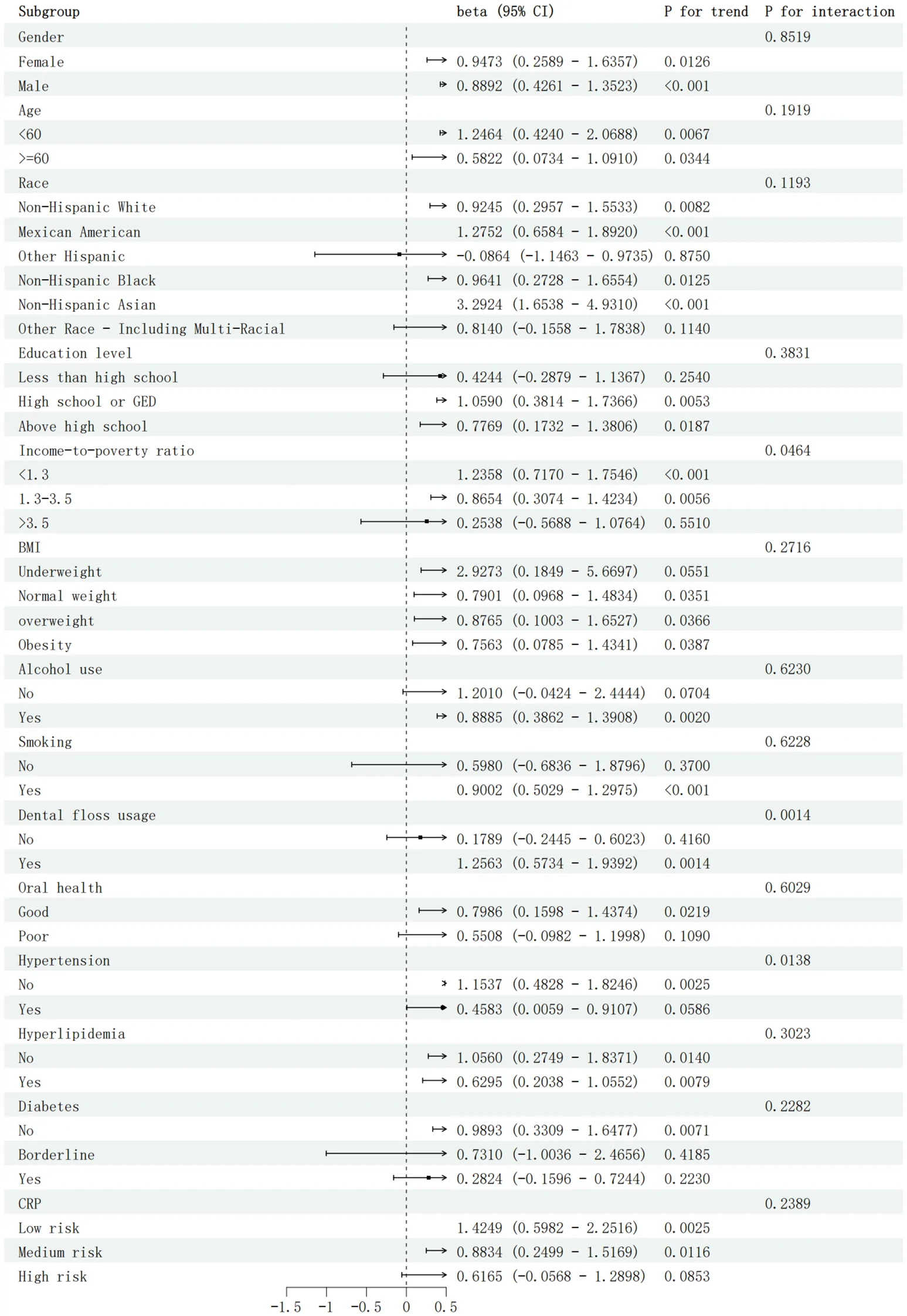
Subgroup analysis of assessing the association between periodontal disease and COPD. (Adjustments were made for a range of demographic and health factors, including age, gender, race, education level, income-to-poverty ratio, BMI, alcohol usage, smoking, dental floss usage, oral health status, hypertension, hyperlipidemia, diabetes, and hsCRP, with the stratification component in question being excluded.).

In **Figure 3**, the outcomes of the stratified analyses and interaction testing are summarized. A considerable positive association between self-reported periodontal disease and COPD was observed in participants both younger than 60 years (*P = 0.0067*) and those aged 60 years or older (*P = 0.0344*). However, a formal test for interaction revealed that age did not significantly modify the relationship (interaction *P = 0.1919*). Significant effect modification was identified for three factors. The link between self-reported periodontal disease and COPD was notably influenced by income level (interaction *P = 0.0464*), hypertension (interaction *P = 0.0138*), and flossing behavior (interaction *P = 0.0014*). No other covariates examined demonstrated statistically significant interaction effects.

### MR analysis investigates the association between periodontal disease and COPD

**Figure 4:**
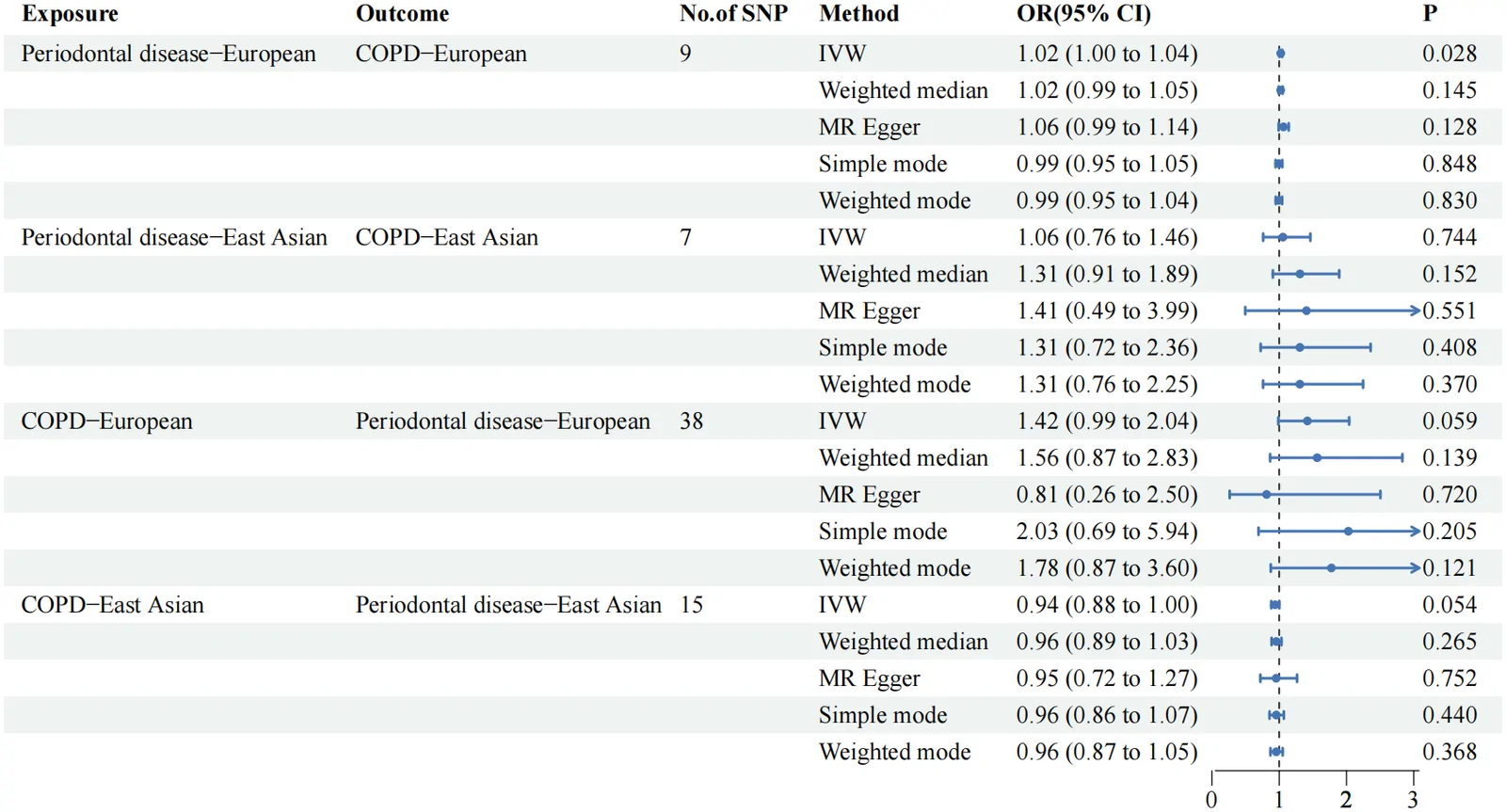
Forest plot of causal effect of COPD and periodontal disease.

Bidirectional two-sample Mendelian randomization analyses were conducted to evaluate the causal link between periodontal disease and COPD across different ancestries. The MR analysis in the European population yielded a modest effect (IVW OR = 1.02, 95% CI: 1.00 to 1.04, *P = 0.028*), which, after Bonferroni correction for multiple comparisons (adjusted significance threshold *P < 0.025* for two ancestries), did not survive strict correction (adjusted *P = 0.056*). Conversely, no evidence supported a reverse causal pathway—from COPD to periodontal disease—in this population (IVW OR = 1.42, 95% CI: 0.99 to 2.04, *P = 0.059*). In the East Asian cohort, no significant causal links were found between periodontal disease and COPD in either direction, with IVW ORs of 1.06 (95% CI: 0.76 to 1.46, *P = 0.744*) for periodontal disease to COPD and 0.94 (95% CI: 0.88 to 1.00, *P = 0.054*) for COPD to periodontal disease (**Figure 4**).

The MR-PRESSO global test detected no significant outliers (global *P > 0.05* for all analyses), and the Steiger directionality test confirmed the assumed direction of causality for the European analysis (Steiger_dir = TRUE, *P = 0.021*); these results are presented in **Supplementary Table 11**. The number of instrumental variables for each analysis was as follows: European periodontal disease → COPD: 9 SNPs; East Asian periodontal disease → COPD: 7 SNPs; European COPD → periodontal disease: 38 SNPs; East Asian COPD → periodontal disease: 15 SNPs (**Supplementary Tables 7**-**S10**). The MR-Egger regression intercept test showed no evidence of directional horizontal pleiotropy (all intercept *P > 0.05*), and Cochran’s Q test indicated no significant heterogeneity among the causal estimates from individual SNPs in either the IVW or MR-Egger models (all *P > 0.05*); both sets of results are presented in **Supplementary Table 12**. Leave-one-out analyses and symmetric funnel plots (**Supplementary Figures 1**–**S4**) revealed no outlying instrumental variables, confirming the robustness and reliability of the causal inference. All SNPs used as instrumental variables had F-statistics > 19, confirming the absence of weak instrument bias (detailed in **Supplementary Tables 7**–**S10**). We also applied Bonferroni correction for multiple comparisons across the two ancestral populations (corrected significance threshold *P < 0.025*). The European MR result remained suggestive but did not survive strict correction (adjusted *P = 0.056*).

### Racial differences in susceptibility to periodontal disease and COPD

Associations between race and the prevalence of self-reported periodontal disease and COPD were examined across four sequentially adjusted models (**Tables 3**, **4**). Across all models — ranging from the unadjusted baseline (Model 1) to those fully adjusted for demographic, socioeconomic, and behavioral covariates (Models 2 – 4)—No significant statistical relationship was detected between race and self-reported periodontal disease prevalence. In marked contrast, a significant racial disparity in COPD susceptibility was evident. Using Non-Hispanic White participants as the reference category, Mexican American and Non-Hispanic Asian individuals consistently exhibited significantly lower odds of COPD across all models (all *P < 0.05*). For Non-Hispanic Black participants, no significant difference relative to the reference group was detected in the crude model (Model 1). However, following adjustment for covariates in Models 2 through 4, Non-Hispanic Black individuals also demonstrated significantly lower susceptibility to COPD compared to Non-Hispanic White individuals (all *P < 0.05*). According to the findings, race is not a major factor in the prevalence of self-reported periodontal disease among this study group, yet it is a key determinant of COPD susceptibility. Non-Hispanic White individuals are significantly more susceptible to COPD compared to Mexican American, Non-Hispanic Asian, and Non-Hispanic Black populations.

**Table 3:** Associations between race and periodontal disease in NHANES 2017-March 2020 Pre-pandemic according to multivariable logistic regression analysis.

Race and CP	Model 1 OR (95% CI) p-value		Model 2 OR (95% CI) p-value		Model 3 OR (95% CI) p-value		Model 4 OR (95% CI) p-value	
Non-hispanic white	Reference		Reference		Reference		Reference	
Mexican American	0.908 (0.628, 1.313)	0.614	0.695 (0.460, 1.051)	0.106	0.726 (0.477, 1.104)	0.161	0.753 (0.505, 1.121)	0.205
Other Hispanic	1.478 (0.978, 2.233)	0.080	1.215 (0.772, 1.911)	0.414	1.303 (0.943, 1.798)	0.287	1.282 (0.794, 2.070)	0.343
Non-Hispanic Black	1.004 (0.769, 1.312)	0.975	0.856 (0.625, 1.171)	0.347	0.891 (0.645, 1.230)	0.497	0.830 (0.596, 1.156)	0.306
Non-Hispanic Asian	0.836 (0.618, 1.130)	0.259	0.813 (0.582, 1.136)	0.245	0.906 (0.633, 1.297)	0.601	0.956 (0.665, 1.376)	0.817
Other Race	1.140 (0.542, 2.396)	0.734	1.004 (0.446, 2.263)	0.992	0.970 (0.434, 2.172)	0.943	0.904 (0.396, 2.066)	0.819

Model 1, Adjusting for age, gender;.

Model 2, Model 1 + adjusted for educational level, ratio of family income to poverty.

Model 3, Model 2 + adjusted for smoking and alcohol use.

Model 4, Model 3+ adjusted for BMI, hypertension, hyperlipidemia, diabetes, and hsCRP.

OR, Odds ratio; CI, Confidence interval.

**Table 4:** Associations between race and COPD in NHANES 2017-March 2020 Pre-pandemic according to multivariable logistic regression analysis.

Race and COPD	Model 1 OR (95% CI) p-value		Model 2 OR (95% CI) p-value		Model 3 OR (95% CI) p-value		Model 4 OR (95% CI) p-value	
Non-hispanic white	Reference		Reference		Reference		Reference	
Mexican American	0.365 (0.232, 0.573)	< 0.001	0.235 (0.153, 0.361)	< 0.001	0.279 (0.179, 0.436)	< 0.001	0.273 (0.169, 0.443)	0.001
Other Hispanic	0.581 (0.323, 1.047)	0.087	0.408 (0.222, 0.750)	0.013	0.538 (0.297, 0.977)	0.071	0.548 (0.310, 0.968)	0.084
Non-Hispanic Black	0.824 (0.591, 1.150)	0.271	0.560 (0.392, 0.800)	0.009	0.631 (0.434, 0.917)	0.044	0.568 (0.390, 0.827)	0.029
Non-Hispanic Asian	0.119 (0.049, 0.293)	< 0.001	0.109 (0.045, 0.260)	< 0.001	0.172 (0.072, 0.410)	0.002	0.196 (0.081, 0.477)	0.008
Other Race	2.041 (1.241, 3.357)	0.012	1.571 (0.959, 2.574)	0.092	1.472 (0.941, 2.304)	0.123	1.339 (0.866, 2.070)	0.250

Model 1, Adjusting for age, gender, race;.

Model 2, Model 1 + adjusted for educational level, ratio of family income to poverty.

Model 3, Model 2 + adjusted for smoking and alcohol use.

Model 4, Model 3+ adjusted for BMI, hypertension, hyperlipidemia, diabetes, and hsCRP.

OR, Odds ratio; CI, Confidence interval.

### Periodontal pathogens induce pro-inflammatory responses in airway epithelial cells

**Figure 5:**
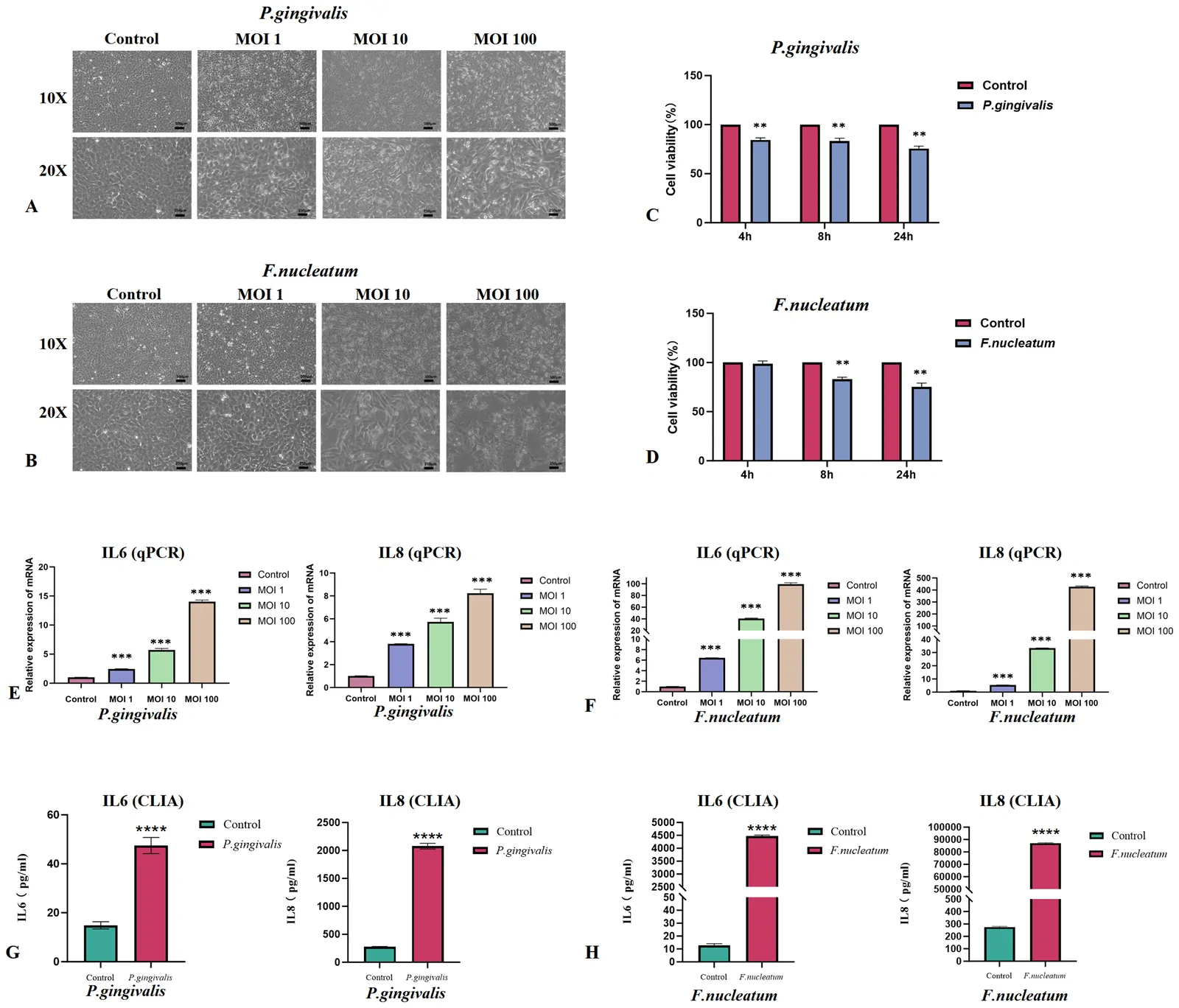
Periodontal pathogens induce pro-inflammatory responses in BEAS-2B cells. **(A)** BEAS-2B cells exhibited reduced cell density and an elongated morphology after co-culture with *P. gingivalis*. **(B)** BEAS-2B cells showed decreased density and an elongated shape following co-culture with *F. nucleatum*. **(C)** Cell viability assessed by CCK-8 assay following treatment with *P. gingivalis* culture supernatant. **(D)** Cell viability assessed by CCK-8 assay following treatment with *F. nucleatum* culture supernatant. **(E)** qPCR analysis of IL-6 and IL-8 mRNA expression in BEAS-2B cells following *P. gingivalis* exposure. **(F)** qPCR analysis of IL-6 and IL-8 mRNA expression following *F. nucleatum* exposure. **(G)** Chemiluminescence immunoassay (CLIA) quantification of IL-6 and IL-8 protein levels in culture supernatants following *P. gingivalis* exposure. **(H)** CLIA quantification of IL-6 and IL-8 protein levels in culture supernatants following *F. nucleatum* exposure. Data are presented as mean ± SD from three independent experiments (n = 3). Statistical significance was determined using one-way ANOVA with Tukey’s post-hoc test. **P < 0.01, ***P < 0.001, ****P < 0.0001 versus control group.

To explore the mechanistic connection between periodontal disease and COPD exacerbation, we initially assessed the direct impact of primary periodontal pathogens on human BEAS-2B cells. Following co-culture with *P. gingivalis*, BEAS-2B cells exhibited notable morphological alterations, including reduced cell density and an elongated phenotype (**Figures 5A, B**). Since live bacteria interfere with the absorbance readings of the CCK-8 assay, we used bacterial culture supernatants rather than live bacteria to avoid this interference. The CCK-8 assay results showed that treatment with *P. gingivalis* culture supernatant reduced cell viability to 75.2 ± 4.1% of the control level, and *F. nucleatum* supernatant reduced it to 77.8 ± 3.6% of the control level (**Figures 5C, D**). qPCR analysis demonstrated that *P. gingivalis* significantly increased mRNA expression of the key pro-inflammatory cytokines IL-6 and IL-8 in a concentration-dependent manner (**Figure 5E**). Specifically, *P. gingivalis* upregulated IL-6 mRNA by 2.45 ± 0.04 fold (*P < 0.01* vs. control) at MOI 1, 5.72 ± 0.25 fold (*P < 0.01*) at MOI 10, and 14.03 ± 0.28 fold (*P < 0.001*) at MOI 100. For IL-8 mRNA, *P. gingivalis* induced increases of 3.81 ± 0.02 fold (*P < 0.01*), 5.74 ± 0.31 fold (*P < 0.01*), and 8.23 ± 0.36 fold (*P < 0.001*) at MOI 1, 10, and 100, respectively. Co-culturing with *F. nucleatum* induced similar cytomorphological alterations and more robustly elevated IL-6 and IL-8 mRNA levels (**Figure 5F**). For IL-6 mRNA, *F. nucleatum* induced increases of 6.43 ± 0.03 fold (*P < 0.001*) at MOI 1, 40.51 ± 0.79 fold (*P < 0.001*) at MOI 10, and 99.27 ± 2.43 fold (*P < 0.001*) at MOI 100. For IL-8 mRNA, *F. nucleatum* induced increases of 5.38 ± 0.03 fold (*P < 0.001*) at MOI 1, 33.32 ± 0.16 fold (*P < 0.001*) at MOI 10, and 427.02 ± 6.28 fold (*P < 0.001*) at MOI 100. Notably, *F. nucleatum* exhibited substantially stronger pro-inflammatory effects than *P. gingivalis* at equivalent MOI conditions. The pathogen-induced cytokine secretion was confirmed at the protein level. CLIA demonstrated that the concentrations of both IL-6 and IL-8 in the culture supernatant were significantly elevated following exposure to either *P. gingivalis* or *F. nucleatum* (**Figures 5G, H**). Specifically, *P. gingivalis* significantly increased IL-6 protein secretion from 14.86 ± 1.49 pg/mL (control) to 47.46 ± 3.31 pg/mL (*P < 0.001*), representing a 3.19 fold increase, and IL-8 protein secretion from 277.33 ± 4.84 pg/mL to 2107.30 ± 47.32 pg/mL (*P < 0.001*), representing a 7.60 fold increase. Notably, *F. nucleatum* exhibited substantially stronger effects at the protein level, elevating IL-6 from 12.80 ± 1.25 pg/mL to 4472.45 ± 36.98 pg/mL (*P < 0.001*), a 349.4 fold increase, and IL-8 from 275.43 ± 5.39 pg/mL to 86996.67 ± 513.43 pg/mL (*P < 0.001*), a 315.9 fold increase. All experiments were performed with three independent replicates (n = 3). Statistical significance was determined using one-way ANOVA with Tukey’s *post-hoc* test.

### Synergistic effect of periodontitis on pulmonary inflammation in a COPD rat model

**Figure 6:**
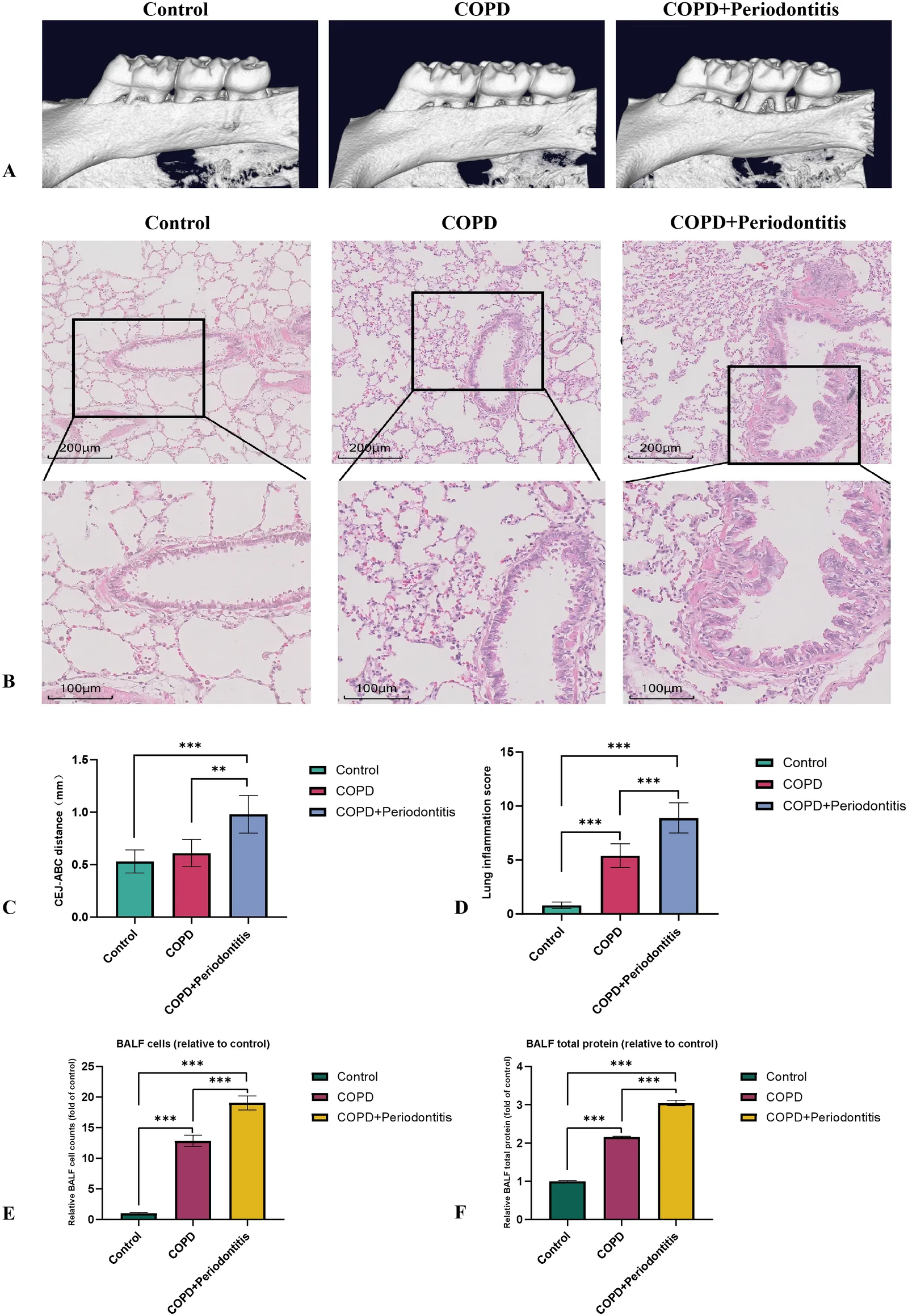
Pre-existing periodontitis exacerbates pulmonary inflammation in a rat COPD model. Rats were assigned to three groups (n = 6 per group). **(A)** Micro-CT images of maxillary alveolar bone (three-dimensional reconstruction). **(B)** H&E-stained lung sections showing inflammatory infiltration, alveolar wall thickening, and structural disruption. **(C)** Quantitative analysis of alveolar bone loss measured by the distance from the cemento-enamel junction to the alveolar bone crest (CEJ-ABC). **(D)** Semi-quantitative pulmonary inflammation scores (0–12). **(E, F)** Relative quantification of BALF total protein concentration and total cell counts, expressed as fold-change relative to the normal control group (set to 1.0). Data are presented as mean ± SD. Statistical significance was determined using one-way ANOVA with Tukey’s post-hoc test. **P < 0.01, ***P < 0.001.

To translate these *in vitro* findings into a pathophysiological context, we assessed lung tissue pathology in a rat model. Hematoxylin and eosin (H&E) staining of lung sections revealed significantly increased inflammatory infiltration, alveolar wall thickening, and structural disruption in rats with both COPD and experimental periodontitis compared to those with only COPD (**Figure 6B**). The semi-quantitative pathological score (**Figure 6D**) showed a clear stepwise increase across the three groups: normal control (0.8 ± 0.3), COPD-only (5.4 ± 1.1), and COPD + periodontitis (8.9 ± 1.4), with all pairwise comparisons reaching statistical significance (*P < 0.001* for all). The relative quantitative results of BALF total cell counts and total protein concentration showed a similar stepwise increasing trend among groups (**Figures 6E, F**). Both parameters were significantly elevated in the COPD and COPD + periodontitis groups compared with the Control group, and were significantly higher in the COPD + periodontitis group than in the COPD group, with all pairwise comparisons reaching statistical significance (all *P < 0.001*). This evidence indicates that pre-existing periodontitis exacerbates pulmonary inflammatory responses in the context of COPD. Micro-CT measurement of maxillary alveolar bone loss (CEJ-ABC distance) confirmed successful periodontitis induction: normal control (0.53 ± 0.11 mm), COPD-only (0.61 ± 0.13 mm, *P > 0.05* vs. normal), and COPD + periodontitis (0.98 ± 0.18 mm, *P < 0.01* vs. both normal and COPD-only) (**Figures 6A, C**).

## Discussion

COPD exhibits a high prevalence among the elderly and is influenced by multiple factors (Cortopassi et al., 2017; Fang et al., 2018). Advancements in periodontal medicine have highlighted the significant impact of oral health on systemic diseases. As a result, increasing research is examining the link between periodontal disease and COPD in older adults (Mendez et al., 2017; Beck et al., 2019). Although numerous studies suggest a correlation, several controversies persist due to variations in sample selection, study design, and measurement methodologies, leading to inconsistent findings (Pérez Barrionuevo et al., 2018; Baldomero et al., 2019; Javaheri et al., 2020). While research shows a higher prevalence of COPD among individuals with periodontal disease, suggesting a potential link, this association may be confounded by shared risk factors like age and smoking rather than representing a direct causal relationship (Bergström et al., 2013). The influence of these confounders, if not sufficiently accounted for, may lead to an overestimation of the association in some observational studies (Hobbins et al., 2017). After adjusting for covariates such as gender, age, race, educational status, poverty income ratio, BMI, smoking, alcohol consumption, hypertension, hyperlipidemia, diabetes, and hsCRP levels, the study found a significant positive association between self-reported periodontal disease and COPD (**Table 2**). However, our findings provide supportive, rather than definitive, evidence for an association between self-reported periodontal disease and COPD. It should also be noted that the NHANES analysis relied on self-reported periodontal disease assessed by a single question (“Do you think you might have gum disease?”), which reflects perceived gum health rather than clinically confirmed periodontitis. This measure is subject to recall and awareness biases, and the absence of clinical periodontal parameters precludes causal interpretation of the cross-sectional associations. Therefore, these results should be interpreted as providing epidemiological associations that require confirmation in prospective cohort studies with clinical measurements.

The causal direction of the observed association, however, requires further elucidation. A study identified a higher prevalence of periodontal disease among COPD patients, but the causality direction is still uncertain (Peter et al., 2013). We used MR analysis on GWAS data to explore this issue. The findings provided suggestive evidence that periodontal disease might be a risk factor for COPD among Europeans. Notably, no significant causal relationship was detected in the East Asian population (**Figure 4**). Furthermore, our evaluation of racial susceptibility indicated no significant association between race and self-reported periodontal disease (**Table 3**), but COPD demonstrated racial differences, with a higher prevalence among Non-Hispanic White people compared to Mexican Americans, Non-Hispanic Asians, and Non-Hispanic Black People (**Table 4**). The MR analysis in the European population yielded a modest effect (IVW OR = 1.02, 95% CI: 1.00 to 1.04, *P = 0.028*), which, after Bonferroni correction for multiple comparisons (adjusted significance threshold *P < 0.025* for two ancestries), did not survive strict correction (adjusted *P = 0.056*). The discrepancy between the European and East Asian MR findings may be explained by multiple factors. A key consideration is statistical power: the European COPD GWAS included 58,559 cases and 937,358 controls (total n = 995,917), whereas the East Asian COPD GWAS comprised only 3,315 cases and 201,592 controls (total n = 204,907)—a more than 17-fold difference in the number of cases, which severely limits the ability to detect modest genetic effects in the East Asian population. Another relevant factor is phenotype definition: the European GWAS were derived from clinically characterized cohorts, whereas the East Asian datasets relied on broader case definitions from biobank-based electronic health records, which may introduce heterogeneity in genetic association signals. Additionally, differences in genetic architecture, allele frequencies, and environmental exposures between populations may modify the exposure-outcome relationship. Given the substantially smaller sample size and limited statistical power of the East Asian datasets, together with the fact that the European MR result did not survive multiple-testing correction (adjusted *P = 0.056*), the MR findings should be interpreted as suggestive and exploratory rather than conclusive. The null finding in East Asians should be interpreted with caution and may reflect insufficient power to detect a modest effect rather than a true absence of causality. Therefore, the MR results should not be generalized to other populations without further investigation.

To explore a potential biological mechanism underlying this association, we investigated the direct effects of key periodontal pathogens. We found *in vitro* experiments that *P. gingivalis* and *F. nucleatum* can upregulate IL-6 and IL-8 in airway epithelial cells, suggesting that periodontal pathogens can directly trigger airway inflammation. Studies have shown that IL-6 and IL-8 are primarily regulated through the NF-κB and p38 MAPK signaling pathways (Zhang et al., 2026). Bacterial components such as lipopolysaccharide (LPS), a major virulence factor of Gram-negative periodontal pathogens including *P. gingivalis*, are known to activate these pathways in epithelial cells (Wang et al., 2020; Zhang et al., 2026). Therefore, we speculate that periodontal pathogens may activate the NF-κB and p38 MAPK signaling pathways in airway epithelial cells through their LPS and other virulence factors, leading to transcriptional upregulation and subsequent secretion of IL-6 and IL-8. In addition to their direct pro-inflammatory effects, these chemokines further promote the recruitment of immune cells, especially neutrophils, into the airway. This recruitment amplifies the local inflammatory response, promoting the occurrence and aggravation of lung inflammation. However, our *in vitro* data indicate activation of airway epithelial inflammation, rather than a COPD specific causal relationship. Although the observed upregulation of IL-6/IL-8 is consistent with the pro-inflammatory phenotype that may promote the pathogenesis of COPD, further research is still needed. Nevertheless, our findings, combined with the known role of NF-κB/p38 MAPK in IL-6/IL-8 regulation and the known functions of these cytokines in recruiting neutrophils and promoting airway inflammation, suggest that periodontal infection may affect COPD susceptibility through the oral pulmonary inflammatory axis.

*In vivo* extension of this mechanism was evaluated using a rat model combining COPD with experimental periodontitis. Compared with the COPD-only group, rats in the combined model exhibited significantly exacerbated pulmonary inflammation, as evidenced by increased inflammatory cell infiltration, pronounced alveolar wall thickening, and marked structural disruption on H&E staining. To further characterize this exacerbation, we performed BALF analysis. The significantly elevated total cell count in BALF indicated substantial recruitment of inflammatory cells into the alveolar space, while the markedly increased total protein level suggested enhanced pulmonary capillary permeability and consequent plasma protein leakage. Collectively, these quantitative findings demonstrate that pre-existing periodontitis synergistically amplifies the pulmonary inflammatory response in the context of COPD. This is consistent with Li et al.’s study, which suggests that periodontal pathogen infection can induce COPD-like pulmonary changes in mouse models, including decreased lung function, increased inflammatory cell infiltration, and elevated levels of tissue damage markers such as matrix metalloproteinase-8 and neutrophil elastase (Li et al., 2024). Furthermore, the synergistic effect may not be confined to the lungs. Emerging evidence suggests that periodontal pathogens can trigger a systemic inflammatory state through complex immunomodulatory mechanisms, affecting multiple organ systems—including the cardiovascular and metabolic systems—and thereby potentially contributing indirectly to the pathological progression of COPD (Mulhall et al., 2020; Hajishengallis and Chavakis, 2021; Lin et al., 2023). *In vitro* experiments demonstrated that *P. gingivalis* and *F. nucleatum* directly activate inflammatory responses in airway epithelial cells in the absence of any mechanical injury. However, these *in vitro* findings cannot substitute for the missing *in vivo* control group. Nevertheless, the lack of a “pathogens-only without ligature” control group in the current study restricts our ability to definitively dissect the relative contributions of mechanical (ligature-induced) inflammation versus pathogen-specific microbial effects to the observed pulmonary aggravation. Future studies should clarify whether the microbial actions of *P. gingivalis* and *F. nucleatum* alone are sufficient to provoke pulmonary inflammation in the absence of mechanical injury, and should incorporate a pathogen-only, non-ligature control group to definitively establish the independent *in vivo* effects of these pathogens.

Given the mechanistic links described above, clinical intervention for periodontal disease holds potential positive significance for COPD management. From a clinical perspective, periodontal treatments (mechanical and pharmacological) may positively impact COPD management, potentially reducing acute exacerbations and improving lung function (Apessos et al., 2021; Kelly et al., 2021). Epidemiological studies indicate a significant negative association between good oral hygiene habit of flossing and COPD (**Table 2**). The interaction test after multiple imputation indicated significant interactions between hypertension (*P = 0. 0138*) and flossing (*P = 0. 0014*) in relation to the connection between self-reported periodontal disease and COPD (**Figure 3**). These interaction findings, while suggestive, should be interpreted with caution given their sensitivity to the missing data approach, and warrant confirmation in future studies with more complete data collection. In our complete-case analysis, flossing frequency and hypertension did not show statistically significant interactions with self-reported periodontal disease in relation to COPD (**Supplementary Figure 5**); however, after multiple imputation, these interactions became statistically significant *(P < 0.05*). This discrepancy likely reflects the inherent trade-offs between the two analytical approaches. Complete-case analysis, by excluding participants with missing data on any of the covariates, reduces sample size and may introduce selection bias if missingness is not completely random. Notably, approximately 19% of the periodontal disease data were missing, and this missingness was disproportionately concentrated among older participants with a higher prevalence of hypertension and less frequent flossing habits. By retaining all available observations, multiple imputation may reduce bias under the missing-at-random assumption and with a correctly specified imputation model, and also helps restore statistical power that would otherwise be lost in complete-case analysis. However, it is important to acknowledge that this assumption cannot be directly verified, and the observed sensitivity of interaction terms to the missing-data method suggests that these findings should be interpreted with caution. Future studies with more complete data collection are warranted to further validate these interaction effects. Exploring the connection between periodontal disease and COPD may offer insights that could inform treatment strategies for COPD patients, highlighting the significance of oral health in overall well-being (Shen et al., 2016; Wang et al., 2024). Future research should prioritize elucidating the causal relationships and underlying mechanisms. High-quality, large-scale prospective cohort and intervention studies are required to manage confounders. Employing multi-omics approaches (genomics, transcriptomics, proteomics, metabolomics) will facilitate a comprehensive analysis of biomarkers and molecular pathways, uncovering potential interaction mechanisms (Guo and Wang, 2025). Further work is needed to explore strategies for integrating periodontal disease prevention and treatment into COPD management. Comprehensive interventions for older adults, including oral hygiene education and synergistic optimization of periodontal and COPD therapies, may represent promising approaches for improving patient quality of life and prognosis. The future management of these conditions will likely become increasingly multidisciplinary and personalized. In managing COPD patients, enhanced communication between respiratory physicians and dental professionals could be beneficial to assess periodontal health and formulate integrated treatment strategies for those with co-morbidities, thereby potentially enhancing outcomes (Dong et al., 2021). Oral hygiene maintenance may represent a supportive strategy that warrants further investigation in COPD management.

International collaboration will play a pivotal role. Multinational, multicenter studies can expand sample sizes and enhance the generalizability of findings. Sharing resources and data across teams will foster a comprehensive exploration of epidemiology, mechanisms, diagnosis, and treatment, accelerating progress (Hajishengallis, 2022). Multidisciplinary integration — encompassing periodontology, pulmonology, microbiology, immunology, and molecular biology (Fischer et al., 2020), is indispensable for understanding the interplay between these diseases. An integrated treatment program for patients with concurrent COPD and periodontitis has been reported to show efficacy in improving both conditions, suggesting potential value for managing this comorbidity in the elderly and possibly improving patient outcomes (Zhou et al., 2014; Shen et al., 2016). The future management of the complex relationship between periodontal disease and COPD will focus on prevention, early intervention, and a multidisciplinary, personalized approach.

## Conclusion

This study integrates epidemiological analysis, Mendelian randomization, *in vitro* experiments, and an *in vivo* animal model to investigate the relationship between periodontal disease and COPD. Our findings suggest that periodontal disease may contribute to COPD susceptibility. *In vitro*, both *P. gingivalis* and *F. nucleatum* directly activate inflammatory responses in airway epithelial cells, supporting the biological plausibility of a direct pathogen-driven mechanism. *In vivo*, pre-existing periodontitis, as a complex disease state involving mechanical injury, plaque accumulation, and polymicrobial dysbiosis—exacerbates pulmonary inflammation in the context of COPD. Together, these complementary findings support the biological plausibility of an oral-lung inflammatory axis, while the independent contribution of specific pathogens to the *in vivo* effects requires further investigation.

However, the reliance on self-reported disease definitions in the NHANES analysis, the modest and ancestry-specific nature of the MR evidence, and the use of simplified *in vitro* and *in vivo* systems that may not fully replicate human disease complexity. Therefore, our results should be interpreted as providing supportive evidence for a link between periodontal health and COPD, rather than definitive proof of causation.

From a translational perspective, the observed associations between oral health status and COPD, suggest that maintaining good oral hygiene, such as regular flossing, may represent a potential low-cost, low-risk adjunctive strategy for COPD management that warrants further investigation. However, given that no intervention was tested in the present study, large-scale prospective cohort studies and randomized controlled trials are needed to confirm whether periodontal interventions can improve COPD outcomes before any clinical recommendations can be made.

## Data Availability

The original contributions presented in the study are included in the article/**Supplementary Material**. Further inquiries can be directed to the corresponding author.
