# Supplementary material for: From oral infection to pulmonary inflammation: periodontal pathogens and their potential role in COPD susceptibility
Source: Front Cell Infect Microbiol. 2026 Aug 28;16:1896617. doi: 10.3389/fcimb.2026.1896617 (PMC13561934; doi:10.3389/fcimb.2026.1896617)
Supplement: Supplementary file 1 [file Supplementaryfile1.docx]

**Article title:** From Oral Infection to Pulmonary Inflammation: Periodontal Pathogens and Their Potential Role in COPD Susceptibility

**Author names:** Xiaohao Liu, Yan Chai, Fengxiang Shi, Bingchun Li, Jiawei Zeng, Jiaming Bi, Shuaimei Xu, Buling Wu

**Affiliation and e-mail address of the corresponding author:** Shenzhen Clinical College of Stomatology, School of Stomatology, Southern Medical University, Shenzhen, 518118, Guangdong Province, China.

Table S1：The baseline characteristics of participants with or without periodontal disease in a cross-sectional study based on the NHANES 2017-March 2020 Pre-pandemic

| **Variables** | **Overall**  **（n=4142）** | **Periodontal disease**  **No（n=3429） Yes（n=713）** | | **P-value** |
| --- | --- | --- | --- | --- |
| COPD, n (%) |  |  |  | < 0.001 |
| Yes | 366 (8.8%) | 256 (7.5%) | 110 (15.4%) |  |
| No | 3776 (91.2%) | 3173 (92.5%) | 603 (84.6%) |  |
| Oral health, n (%) |  |  |  | < 0.001 |
| Good | 2758 (66.6%) | 2581 (75.3%) | 177 (24.8%) |  |
| Poor | 1384 (33.4%) | 848 (24.7%) | 536 (75.2%) |  |
| Age, Mean±SD | 49.46±18.29 | 48.72±19.01 | 53.03±13.84 | < 0.001 |
| Age strata, n (%) |  |  |  | 0.857 |
| ＜60 | 2704 (65.3%) | 2240 (65.3%) | 464 (65.1%) |  |
| ≥60 | 1438 (34.7%) | 1189 (34.7%) | 249 (34.9%) |  |
| Gender, n (%) |  |  |  | 0.185 |
| Male | 1991 (48.1%) | 1634 (47.7%) | 357 (50.1%) |  |
| Female | 2151 (51.9%) | 1795 (52.3%) | 356 (49.9%) |  |
| Race, n (%) |  |  |  | 0.304 |
| Non-Hispanic White | 1405 (33.9%) | 1160 (33.8%) | 245 (34.4%) |  |
| Mexican American | 505 (12.2%) | 421 (12.3%) | 84 (11.8%) |  |
| Other Hispanic | 399 (9.6%) | 321 (9.4%) | 78 (10.9%) |  |
| Non-Hispanic Black | 1093 (26.4%) | 902 (26.3%) | 191 (26.8%) |  |
| Non-Hispanic Asian | 517 (12.5%) | 441 (12.9%) | 76 (10.7%) |  |
| Other Race | 223 (5.4%) | 184 (5.4%) | 39 (5.5%) |  |
| Education level, n (%) |  |  |  | < 0.001 |
| Under high school | 760 (18.3%) | 608 (17.7%) | 152 (21.3%) |  |
| High school/GED or equivalent | 1004 (24.2%) | 828 (24.1%) | 176 (24.7%) |  |
| Above high school | 2378 (57.4%) | 1993 (58.1%) | 385 (54.0%) |  |
| Income-to-poverty ratio, n (%) |  |  |  | 0.013 |
| ＜1.3 | 1156 (27.9%) | 923 (26.9%) | 233 (32.7%) |  |
| 1.3-3.5 | 1753 (42.3%) | 1463 (42.7%) | 290 (40.7%) |  |
| ＞3.5 | 1233 (29.8%) | 1043 (30.4%) | 190 (26.6%) |  |
| Alcohol use, n (%) |  |  |  | 0.490 |
| Yes | 3763 (90.8%) | 3099 (90.4%) | 664 (93.1%) |  |
| No | 379 (9.2%) | 330 (9.6%) | 49 (6.9%) |  |
| Smoking, n (%) |  |  |  | 0.005 |
| Yes | 1687 (40.7%) | 1336 (39.0%) | 351 (49.2%) |  |
| No | 2455 (59.3%) | 2093 (61.0%) | 362 (50.8%) |  |
| Dental floss usage |  |  |  | < 0.001 |
| Yes | 3016 (72.8%) | 2534 (73.9%) | 482 (67.6%) |  |
| No | 1126 (27.2%) | 895 (26.1%) | 231 (32.4%) |  |
| BMI, n (%) |  |  |  | 0.209 |
| Underweight | 72 (1.7%) | 56 (1.6%) | 16 (2.2%) |  |
| Normal weight | 1051 (25.4%) | 896 (26.1%) | 155 (21.7%) |  |
| Overweight | 1318 (31.8%) | 1101 (32.1%) | 217 (30.4%) |  |
| Obesity | 1701 (41.1%) | 1376 (40.1%) | 325 (45.6%) |  |
| Hypertension, n (%) |  |  |  | < 0.001 |
| Yes | 1552 (37.5%) | 1216 (35.5%) | 336 (47.1%) |  |
| No | 2590 (62.5%) | 2213 (64.5%) | 377 (52.9%) |  |
| Hyperlipidemia, n (%) |  |  |  | < 0.001 |
| Yes | 1455 (35.1%) | 1151 (33.6%) | 304 (42.6%) |  |
| No | 2687 (64.9%) | 2278 (66.4%) | 409 (57.4%) |  |
| Diabetes, n (%) |  |  |  | < 0.001 |
| Yes | 636 (15.4%) | 480 (14.0%) | 156 (21.9%) |  |
| Borderline | 123 (3.0%) | 92 (2.7%) | 31 (4.3%) |  |
| No | 3383 (81.7%) | 2857 (83.3%) | 526 (73.8%) |  |
| Fasting Glucose (mmol/L), Mean±SD | 6.24±2.01 | 6.18±1.94 | 6.53±2.32 | 0.004 |
| Glycohemoglobin (%), Mean±SD | 5.84±1.11 | 5.80±1.07 | 6.02±1.29 | < 0.001 |
| hsCRP（mg/L）, Mean±SD | 4.01±7.38 | 3.82±6.84 | 4.89±9.52 | 0.051 |
| hsCRP（mg/L）, n (%) |  |  |  | 0.166 |
| Low risk | 1250 (30.2%) | 1070 (31.2%) | 180 (25.2%) |  |
| Medium risk | 1394 (33.7%) | 1167 (34.0%) | 227 (31.8%) |  |
| High risk | 1498 (36.2%) | 1192 (34.8%) | 306 (42.9%) |  |

Table S2：Missing rate, Mean R-hat and Variance R-hat for each variable

| **Variables** | **Sample size (N)** | **Missing rate (Missing %)** | **Mean R-hat** | **Variance R-hat** |
| --- | --- | --- | --- | --- |
| COPD | 3963 | 4.32% | 1.02 | 1.02 |
| Periodontal disease | 3368 | 18.69% | 1.01 | 1.01 |
| Oral health | 4142 | 0 | NA | NA |
| Age | 4142 | 0 | NA | NA |
| Gender | 4142 | 0 | NA | NA |
| Race | 4142 | 0 | NA | NA |
| Education leve | 3963 | 4.32% | 1.01 | 1.00 |
| Income-to-poverty ratio | 3657 | 11.71% | 1.00 | 1.00 |
| Alcohol use | 3892 | 6.04% | 1.01 | 1.01 |
| Smoking | 4142 | 0 | NA | NA |
| Dental floss usage | 3368 | 18.69% | 1.01 | 1.00 |
| BMI | 4059 | 2.00% | 1.00 | 1.01 |
| Hypertension | 4142 | 0 | NA | NA |
| Hyperlipidemia | 4142 | 0 | NA | NA |
| Diabetes | 4142 | 0 | NA | NA |
| Fasting Glucose | 3893 | 6.01% | 1.07 | 1.08 |
| Glycohemoglobin | 3923 | 5.29% | 1.07 | 1.09 |
| hsCRP | 3813 | 7.94% | 1.00 | 1.00 |

Missing rates are calculated as (number of missing cases / total sample size) × 100%. Missing values were imputed using multiple imputation by chained equations (MICE). R-hat: potential scale reduction factor). NA indicates variables with no missing data, for which R-hat is not applicable.

Table S3：Complete Case Analysis Results - Associations between periodontal disease and COPD in NHANES 2017-March 2020 Pre-pandemic according to multivariable logistic regression analysis.

| **Variables** | **Model 1 OR (95% CI) P-value** | | **Model 2 OR (95% CI) P-value** | | **Model 3 OR (95% CI) P-value** | | **Model 4 OR (95% CI) P-value** | |
| --- | --- | --- | --- | --- | --- | --- | --- | --- |
| Periodontal disease | 1.850 (1.176, 2.911) | 0.016 | 1.598 (1.050, 2.431) | 0.048 | 1.546 (1.049, 2.278) | 0.049 | 1.445 (0.990, 2.109) | 0.105 |
| Oral health | 2.274 (1.452, 3.561) | 0.002 | 1.665 (0.998, 2.776) | 0.073 | 1.518 (0.910, 2.535) | 0.138 | 1.431 (0.881, 2.322) | 0.198 |
| Dental floss usage | 0.341 (0.268, 0.433) | < 0.001 | 0.451 (0.360, 0.565) | < 0.001 | 0.461 (0.364, 0.584) | < 0.001 | 0.451 (0.356, 0.571) | < 0.001 |

Model 1: Adjusting for age, gender, race;

Model 2: Model 1 + adjusted for educational level, ratio of family income to poverty.

Model 3: Model 2 + adjusted for smoking and alcohol use.

Model 4: Model 3+ adjusted for BMI, hypertension, hyperlipidemia, diabetes, and hsCRP.

OR: Odds ratio; CI: Confidence interval.

Table S4：Complete Case Analysis Results - Associations between race and periodontal disease in NHANES 2017-March 2020 Pre-pandemic according to multivariable logistic regression analysis.

| **Race and CP** | **Model 1 OR (95% CI) P-value** | | **Model 2 OR (95% CI) P-value** | | **Model 3 OR (95% CI) P-value** | | **Model 4 OR (95% CI) P-value** | |
| --- | --- | --- | --- | --- | --- | --- | --- | --- |
| **Non-Hispanic White** | **Reference** |  | **Reference** |  | **Reference** |  | **Reference** |  |
| Mexican American | 0.952 (0.625, 1.452) | 0.825 | 0.679 (0.433, 1.065) | 0.114 | 0.702 (0.437, 1.128) | 0.169 | 0.711 (0.449, 1.127) | 0.190 |
| Other Hispanic | 1.459 (0.898, 2.370) | 0.145 | 1.163 (0.688, 1.967) | 0.581 | 1.208 (0.699, 2.086) | 0.512 | 1.194 (0.675, 2.112) | 0.561 |
| Non-Hispanic Black | 1.085 (0.766, 1.536) | 0.651 | 0.883 (0.615, 1.269) | 0.513 | 0.890 (0.614, 1.319) | 0.599 | 0.825 (0.550, 1.238) | 0.385 |
| Non-Hispanic Asian | 1.039 (0.718, 1.504) | 0.841 | 1.034 (0.692, 1.546) | 0.872 | 1.045 (0.666, 1.639) | 0.851 | 1.069 (0.938, 1.219) | 0.771 |
| Other Race | 1.074 (0.452, 2.555) | 0.873 | 0.883 (0.325, 2.398) | 0.811 | 0.872 (0.326, 2.331) | 0.789 | 0.807 (0.294, 2.214) | 0.690 |

Model 1: Adjusting for age, gender;

Model 2: Model 1 + adjusted for educational level, ratio of family income to poverty.

Model 3: Model 2 + adjusted for smoking and alcohol use.

Model 4: Model 3+ adjusted for BMI, hypertension, hyperlipidemia, diabetes, and hsCRP. .

OR: Odds ratio; CI: Confidence interval;

Table S5：Complete Case Analysis Results - Associations between race and COPD in NHANES 2017-March 2020 Pre-pandemic according to multivariable logistic regression analysis.

| **Race and COPD** | **Model 1 OR (95% CI) P-value** | | **Model 2 OR (95% CI) P-value** | | **Model 3 OR (95% CI) P-value** | | **Model 4 OR (95% CI) P-value** | |
| --- | --- | --- | --- | --- | --- | --- | --- | --- |
| **Non-Hispanic White** | **Reference** |  | **Reference** |  | **Reference** |  | **Reference** |  |
| Mexican American | 0.252 (0.149, 0.425) | < 0.001 | 0.158 (0.094, 0.266) | < 0.001 | 0.194 (0.118, 0.319) | < 0.001 | 0.182 (0.101, 0.325) | < 0.001 |
| Other Hispanic | 0.561 (0.325, 0.967) | 0.052 | 0.395 (0.224, 0.696) | 0.006 | 0.518 (0.303, 0.888) | 0.034 | 0.518 (0.305, 0.879) | 0.044 |
| Non-Hispanic Black | 0.766 (0.561, 1.044) | 0.109 | 0.520 (0.374, 0.723) | 0.002 | 0.581 (0.401, 0.842) | 0.014 | 0.492 (0.336, 0.721) | 0.008 |
| Non-Hispanic Asian | 0.122 (0.033, 0.447) | 0.005 | 0.119 (0.033, 0.419) | 0.005 | 0.182 (0.052, 0.638) | 0.021 | 0.197 (0.054, 0.711) | 0.042 |
| Other Race | 2.177 (1.187, 3.995) | 0.022 | 1.520 (0.817, 2.829) | 0.208 | 1.537 (0.896, 2.636) | 0.144 | 1.384 (0.789, 2.429) | 0.295 |

Model 1: Adjusting for age, gender, race;

Model 2: Model 1 + adjusted for educational level, ratio of family income to poverty.

Model 3: Model 2 + adjusted for smoking and alcohol use.

Model 4: Model 3+ adjusted for BMI, hypertension, hyperlipidemia, diabetes, and hsCRP.

OR: Odds ratio; CI: Confidence interval.

Table S6：Detailed information on GWAS summary statistics used in MR analysis

| Exposures/Outcomes name | Consortium | Ethnicity | Sample sizes | N_Cases | N_Controls | Year | Reference |
| --- | --- | --- | --- | --- | --- | --- | --- |
| COPD-European | Catalog GWAS | European | 995917 | 58,559 | 937,358 | 2022 | PMID：36777996 |
| Periodontal disease-European | Catalog GWAS | European | 9693 | 3569 | 6124 | 2016 | PMID：26962152 |
| COPD-East Asian | NBDC | East Asian | 204907 | 3315 | 201592 | 2020 | PMID：32514122 |
| Periodontal disease-East Asian | Catalog GWAS | East Asian | 178726 | 9560 | 169,166 | 2021 | PMID：34594039 |

GWAS：Genome-wide association study；NBDC：National bioscience database centre；

Table S7： Independent SNPs associated with Periodontal disease-European in the GWAS database.

| SNP | Beta | SE | p | Sample size | Effect_allele | Other_allele | EAF | F | R^2^ |
| --- | --- | --- | --- | --- | --- | --- | --- | --- | --- |
| rs10232172 | 0.45298 | 0.08112 | 2.35E-08 | 9693 | G | T | 0.07807 | 31.18188 | 0.02954 |
| rs10265728 | -0.37192 | 0.08236 | 6.31E-06 | 9693 | A | C | 0.07098 | 20.39052 | 0.01824 |
| rs1111259 | 0.34956 | 0.07729 | 6.11E-06 | 9693 | C | T | 0.09595 | 20.45214 | 0.02120 |
| rs11536897 | 0.46701 | 0.10257 | 5.29E-06 | 9693 | A | G | 0.04566 | 20.72933 | 0.01901 |
| rs1740371 | 0.54935 | 0.11675 | 2.53E-06 | 9693 | G | T | 0.94640 | 22.14041 | 0.03062 |
| rs321224 | -0.35845 | 0.07828 | 4.67E-06 | 9693 | C | T | 0.90200 | 20.96719 | 0.02271 |
| rs7144384 | 0.65630 | 0.12386 | 1.17E-07 | 9693 | A | G | 0.04848 | 28.07657 | 0.03974 |
| rs9915618 | -0.25588 | 0.05412 | 2.26E-06 | 9693 | A | C | 0.26610 | 22.35732 | 0.02557 |
| rs9980417 | 0.49825 | 0.10043 | 7.01E-07 | 9693 | A | C | 0.04741 | 24.61298 | 0.02242 |

EAF, effect allele frequency

Table S8：Independent SNPs associated with Periodontal disease-East Asian in the GWAS database.

| SNP | Beta | SE | p | Sample size | Effect_allele | Other_allele | EAF | F | R^2^ |
| --- | --- | --- | --- | --- | --- | --- | --- | --- | --- |
| rs12632355 | 0.09407 | 0.02024 | 3.35E-06 | 178726 | T | C | 0.83040 | 21.60475 | 0.00249 |
| rs1374625 | 0.08177 | 0.01749 | 2.92E-06 | 178726 | C | T | 0.75373 | 21.86613 | 0.00248 |
| rs150011945 | 0.35371 | 0.07525 | 2.60E-06 | 178726 | G | A | 0.98437 | 22.09183 | 0.00385 |
| rs4847159 | -0.08653 | 0.01772 | 1.05E-06 | 178726 | C | T | 0.76680 | 23.83546 | 0.00268 |
| rs4859880 | 0.06795 | 0.01522 | 8.04E-06 | 178726 | A | G | 0.42517 | 19.92919 | 0.00226 |
| rs7609775 | -0.07107 | 0.01590 | 7.82E-06 | 178726 | G | T | 0.65994 | 19.98198 | 0.00227 |
| rs9408824 | -0.07374 | 0.01620 | 5.30E-06 | 178726 | G | A | 0.32095 | 20.72721 | 0.00237 |

EAF, effect allele frequency

Table S9：Independent SNPs associated with COPD-European in the GWAS database.

| SNP | Beta | SE | p | Sample size | Effect_allele | Other_allele | EAF | F | R^2^ |
| --- | --- | --- | --- | --- | --- | --- | --- | --- | --- |
| rs10057395 | -0.03311 | 0.00671 | 7.95E-07 | 995917 | G | A | 0.54620 | 24.37178 | 0.00054 |
| rs10089406 | 0.03470 | 0.00726 | 1.74E-06 | 995917 | C | T | 0.61540 | 22.86676 | 0.00057 |
| rs1023518 | 0.05268 | 0.00746 | 1.66E-12 | 995917 | T | G | 0.28400 | 49.84658 | 0.00113 |
| rs10473354 | 0.04371 | 0.00831 | 1.42E-07 | 995917 | G | A | 0.38580 | 27.69863 | 0.00091 |
| rs10519237 | -0.06821 | 0.01465 | 3.21E-06 | 995917 | T | C | 0.05342 | 21.68887 | 0.00047 |
| rs10838160 | -0.03925 | 0.00823 | 1.84E-06 | 995917 | G | A | 0.40300 | 22.75436 | 0.00074 |
| rs10883993 | -0.04021 | 0.00756 | 1.06E-07 | 995917 | A | G | 0.31120 | 28.26609 | 0.00069 |
| rs11001397 | -0.03013 | 0.00675 | 8.15E-06 | 995917 | T | C | 0.55940 | 19.90415 | 0.00045 |
| rs11038812 | 0.03493 | 0.00735 | 1.99E-06 | 995917 | A | G | 0.28960 | 22.61060 | 0.00050 |
| rs11174501 | 0.07465 | 0.01425 | 1.60E-07 | 995917 | A | C | 0.05673 | 27.46125 | 0.00060 |
| rs11208766 | -0.03386 | 0.00668 | 4.03E-07 | 995917 | A | G | 0.48820 | 25.67809 | 0.00057 |
| rs11928675 | -0.03450 | 0.00755 | 4.92E-06 | 995917 | T | C | 0.26710 | 20.86642 | 0.00047 |
| rs1246642 | 0.04165 | 0.00795 | 1.62E-07 | 995917 | T | C | 0.76350 | 27.44348 | 0.00063 |
| rs12770228 | 0.03306 | 0.00712 | 3.40E-06 | 995917 | A | G | 0.32090 | 21.57542 | 0.00048 |
| rs13141641 | -0.06407 | 0.00677 | 2.92E-21 | 995917 | C | T | 0.45270 | 89.59857 | 0.00203 |
| rs1322187 | 0.04507 | 0.00788 | 1.08E-08 | 995917 | T | G | 0.32530 | 32.70094 | 0.00089 |
| rs17409597 | 0.04551 | 0.00743 | 8.89E-10 | 995917 | C | T | 0.49340 | 37.55444 | 0.00104 |
| rs1900009 | 0.04041 | 0.00865 | 2.96E-06 | 995917 | A | G | 0.18380 | 21.83978 | 0.00049 |
| rs1950443 | 0.04207 | 0.00917 | 4.50E-06 | 995917 | A | G | 0.21120 | 21.04115 | 0.00059 |
| rs1996664 | 0.05249 | 0.00965 | 5.41E-08 | 995917 | G | A | 0.15590 | 29.56315 | 0.00073 |
| rs2090374 | 0.03745 | 0.00833 | 6.88E-06 | 995917 | T | C | 0.20180 | 20.22564 | 0.00045 |
| rs2160203 | -0.04447 | 0.00765 | 6.18E-09 | 995917 | G | A | 0.25860 | 33.77704 | 0.00076 |
| rs2221298 | -0.03672 | 0.00685 | 8.15E-08 | 995917 | T | C | 0.60340 | 28.77040 | 0.00065 |
| rs2601791 | 0.03643 | 0.00729 | 5.77E-07 | 995917 | G | A | 0.58520 | 24.98628 | 0.00064 |
| rs2760260 | -0.04041 | 0.00772 | 1.65E-07 | 995917 | T | C | 0.67810 | 27.40443 | 0.00071 |
| rs323598 | 0.03967 | 0.00712 | 2.53E-08 | 995917 | T | C | 0.35780 | 31.03711 | 0.00072 |
| rs4423631 | 0.06311 | 0.01144 | 3.44E-08 | 995917 | C | T | 0.82820 | 30.44215 | 0.00113 |
| rs4442537 | -0.03139 | 0.00680 | 3.86E-06 | 995917 | T | C | 0.41030 | 21.33313 | 0.00048 |
| rs4710465 | -0.03560 | 0.00743 | 1.64E-06 | 995917 | T | C | 0.28220 | 22.97351 | 0.00051 |
| rs4946258 | 0.03640 | 0.00720 | 4.30E-07 | 995917 | T | C | 0.30930 | 25.55440 | 0.00057 |
| rs4950953 | 0.03292 | 0.00741 | 8.76E-06 | 995917 | C | T | 0.46270 | 19.76376 | 0.00054 |
| rs6446731 | 0.04115 | 0.00728 | 1.58E-08 | 995917 | A | G | 0.29420 | 31.94633 | 0.00070 |
| rs6504154 | 0.05858 | 0.01286 | 5.23E-06 | 995917 | A | G | 0.07322 | 20.75135 | 0.00047 |
| rs7002282 | -0.03969 | 0.00699 | 1.34E-08 | 995917 | G | A | 0.58310 | 32.27559 | 0.00077 |
| rs7130588 | 0.03849 | 0.00705 | 4.73E-08 | 995917 | G | A | 0.33420 | 29.82443 | 0.00066 |
| rs7173826 | -0.03966 | 0.00710 | 2.32E-08 | 995917 | G | T | 0.33210 | 31.20541 | 0.00070 |
| rs8040868 | 0.10658 | 0.00684 | 9.00E-55 | 995917 | C | T | 0.38770 | 242.91545 | 0.00539 |
| rs8047812 | 0.03373 | 0.00734 | 4.28E-06 | 995917 | G | A | 0.64530 | 21.13573 | 0.00052 |

EAF, effect allele frequency

Table S10：Independent SNPs associated with COPD-East Asian in the GWAS database.

| SNP | Beta | SE | p | Sample size | Effect_allele | Other_allele | EAF | F | R^2^ |
| --- | --- | --- | --- | --- | --- | --- | --- | --- | --- |
| rs1289389 | -0.12568 | 0.02810 | 7.72E-06 | 204907 | T | C | 0.28153 | 20.00597 | 0.00639 |
| rs13137163 | -0.14518 | 0.03260 | 8.45E-06 | 204907 | C | A | 0.18995 | 19.83307 | 0.00649 |
| rs1468131 | 0.22951 | 0.03697 | 5.36E-10 | 204905 | A | C | 0.18876 | 38.54246 | 0.01613 |
| rs1529672 | -0.15305 | 0.02651 | 7.74E-09 | 204907 | A | C | 0.34170 | 33.33932 | 0.01054 |
| rs1585258 | 0.15865 | 0.02678 | 3.13E-09 | 204907 | T | G | 0.65133 | 35.10350 | 0.01143 |
| rs28752520 | -0.17122 | 0.03513 | 1.10E-06 | 204907 | C | T | 0.16046 | 23.75238 | 0.00790 |
| rs3864302 | -0.13329 | 0.02714 | 9.10E-07 | 204907 | T | C | 0.32408 | 24.11002 | 0.00778 |
| rs4261773 | 0.19751 | 0.04259 | 3.53E-06 | 204907 | T | C | 0.12453 | 21.50669 | 0.00851 |
| rs4709558 | -0.13948 | 0.02854 | 1.03E-06 | 204907 | G | A | 0.27313 | 23.87689 | 0.00772 |
| rs56129017 | 0.25712 | 0.03044 | 3.00E-17 | 204907 | T | C | 0.26574 | 71.34599 | 0.02580 |
| rs59513878 | 0.11366 | 0.02537 | 7.48E-06 | 204907 | T | G | 0.51133 | 20.06646 | 0.00646 |
| rs6732115 | 0.16240 | 0.03588 | 6.00E-06 | 204907 | C | T | 0.14527 | 20.48773 | 0.00655 |
| rs7255985 | -0.19926 | 0.03404 | 4.79E-09 | 204907 | T | C | 0.16930 | 34.27171 | 0.01117 |
| rs7299183 | 0.20188 | 0.02892 | 2.95E-12 | 204905 | A | G | 0.62957 | 48.71847 | 0.01901 |
| rs8036417 | 0.14583 | 0.03198 | 5.12E-06 | 204907 | G | A | 0.19079 | 20.79114 | 0.00657 |

EAF, effect allele frequency

Table S11：MR‑PRESSO global test and Steiger directionality test results.

| **Exposure → Outcome (Population)** | **MR‑PRESSO Global P‑value** | **Outlier SNPs (P < 0.05)** | **Steiger**  **P‑value** | **Steiger Direction Confirmed** |
| --- | --- | --- | --- | --- |
| Periodontal disease → COPD (European) | 0.342 | None | 0.021 | Yes |
| Periodontal disease → COPD (East Asian) | 0.621 | None | 0.084 | Not significant |
| COPD → Periodontal disease (European) | 0.458 | None | 0.072 | Not significant |
| COPD → Periodontal disease (East Asian) | 0.552 | None | 0.091 | Not significant |

Table S12：Heterogeneity and pleiotropy tests for Mendelian randomization analyses.

| Exposure | Outcome | heterogeneity | | | | Pleiotropy_test | | |
| --- | --- | --- | --- | --- | --- | --- | --- | --- |
|  |  |  | Q | Q_df | p | Egger_intercept | SE | p |
| Periodontal disease-European | COPD-European | MR Egger | 6.84956 | 7 | 0.44471 | -0.01663 | 0.01458 | 0.29168 |
|  |  | Inverse variance weighted | 8.14979 | 8 | 0.41898 |  |  |  |
| Periodontal disease-East Asian | COPD-East Asian | MR Egger | 8.37668 | 5 | 0.13666 | -0.02577 | 0.04525 | 0.59365 |
|  |  | Inverse variance weighted | 8.92004 | 6 | 0.17812 |  |  |  |
| COPD-European | Periodontal disease-European | MR Egger | 28.95822 | 36 | 0.79135 | 0.02646 | 0.02581 | 0.31224 |
|  |  | Inverse variance weighted | 30.00869 | 37 | 0.78568 |  |  |  |
| COPD-East Asian | Periodontal disease-East Asian | MR Egger | 20.02257 | 13 | 0.09465 | -0.00284 | 0.02442 | 0.90911 |
|  |  | Inverse variance weighted | 20.04344 | 14 | 0.12878 |  |  |  |

Table S13：Semi-quantitative scoring criteria for pulmonary inflammation on H&E stained.

| **Score** | **Alveolar Wall Thickening** | **Inflammatory Cell Infiltration** | **Alveolar Structural Disruption** |
| --- | --- | --- | --- |
| **0** | Alveolar septa are thin and delicate, with no evidence of thickening | No inflammatory cells observed in the alveolar septa or spaces | Alveolar architecture is completely intact with normal airspace morphology |
| **1** | Septa show mild thickening (< 2‑fold compared to normal), with minimal extracellular matrix deposition | Scattered inflammatory cells (< 10% of the visual field), predominantly mononuclear, with no aggregation | Minimal focal disruption affecting < 10% of alveoli, with preserved overall architecture |
| **2** | Septa exhibit moderate thickening (2–3‑fold), with extracellular matrix deposition and early fibrotic changes | Focal aggregates of inflammatory cells (10–30% of the visual field), mainly mononuclear and occasional neutrophils | Moderate structural disruption affecting 10–30% of alveoli, with some airspace obliteration |
| **3** | Septa display severe thickening (> 3‑fold), with dense extracellular matrix deposition and reticular fiber proliferation | Diffuse infiltration (30–60% of the visual field) with mixed inflammatory cells (mononuclear and neutrophils), forming patches of consolidation | Significant disruption affecting 30–50% of alveoli, with visible alveolar collapse or fusion |
| **4** | Diffuse thickening with complete loss of normal septal architecture and extensive fibrosis | Severe diffuse infiltration (> 60% of the visual field) with dense inflammatory cell accumulation, forming extensive areas of consolidation | Extensive disruption involving > 50% of alveoli, with widespread alveolar collapse, fusion, or replacement by inflammatory tissue |

Figure S1. Causal effects of Periodontal disease-European on COPD-European. A: Scatter plot of Tuberculosis effect estimates on COPD. B: Forest plot summarizing Tuberculosis’s overall impact on COPD. C: Funnel plot for bias assessment of the estimates. D: Sensitivity analysis via “leave-one-out” plots.

Figure S2. Causal effects of Periodontal disease-East Asian on COPD-East Asian. A: Scatter plot of Tuberculosis effect estimates on COPD. B: Forest plot summarizing Tuberculosis’s overall impact on COPD. C: Funnel plot for bias assessment of the estimates. D: Sensitivity analysis via “leave-one-out” plots.

Figure S3. Causal effects of COPD-European on Periodontal disease-European. A: Scatter plot of Tuberculosis effect estimates on COPD. B: Forest plot summarizing Tuberculosis’s overall impact on COPD. C: Funnel plot for bias assessment of the estimates. D: Sensitivity analysis via “leave-one-out” plots.

Figure S4. Causal effects of COPD-East Asian on Periodontal disease-East Asian. A: Scatter plot of Tuberculosis effect estimates on COPD. B: Forest plot summarizing Tuberculosis’s overall impact on COPD. C: Funnel plot for bias assessment of the estimates. D: Sensitivity analysis via “leave-one-out” plots.

Figure S5：Complete Case Analysis Results - subgroup analysis of assessing the association between periodontal disease and COPD.

Figure S6：Trace plots of the MICE algorithm for key imputed variables. The figure displays the mean (left panel) and standard deviation (right panel) of imputed values across iterations for each imputed variable. Each colored line represents one of the 20 imputed datasets. After 20 iterations, the streams intermingle and are free of any trend, indicating that the algorithm had adequately converged.

Figure S7：Density plots comparing observed and imputed distributions. Blue curves represent the distribution of observed values; red curves represent the distribution of imputed values. The imputed distributions closely overlap with the observed distributions, indicating that the imputation model produced plausible values.
